# Annexin A1 sustains tumor metabolism and cellular proliferation upon stable loss of HIF1A

**DOI:** 10.18632/oncotarget.6793

**Published:** 2015-12-29

**Authors:** Nadine Rohwer, Fabian Bindel, Christina Grimm, Suling J. Lin, Jessica Wappler, Bertram Klinger, Nils Blüthgen, Ilona Du Bois, Bernd Schmeck, Hans Lehrach, Marjo de Graauw, Emanuel Goncalves, Julio Saez-Rodriguez, Patrick Tan, Heike I. Grabsch, Alessandro Prigione, Stefan Kempa, Thorsten Cramer

**Affiliations:** ^1^ Hepatologie und Gastroenterologie, Campus Virchow-Klinikum, Charité, Berlin, Germany; ^2^ German Cancer Consortium (DKTK), Heidelberg, Germany; ^3^ German Cancer Research Center (DKFZ), Heidelberg, Germany; ^4^ Berlin Institute for Medical Systems Biology, Max-Delbrück-Center for Molecular Medicine, Berlin, Germany; ^5^ Max-Planck-Institut for Molecular Genetics, Berlin, Germany; ^6^ Duke-NUS Graduate Medical School, Singapore; ^7^ Institute of Pathology, Charité - Universitätsmedizin Berlin, Berlin, Germany; ^8^ Integrative Research Institute (IRI) for the Life Sciences and Institute for Theoretical Biology, Humboldt-Universität Berlin, Berlin, Germany; ^9^ Institute for Lung Research, Universities of Giessen and Marburg Lung Center, Philipps-University, Marburg, Germany; ^10^ Division of Toxicology, Leiden/Amsterdam Center for Drug Research, Leiden University, Amsterdam, The Netherlands; ^11^ European Molecular Biology Laboratory, European Bioinformatics Institute (EMBL-EBI), Wellcome Trust Genome Campus, Cambridge, United Kingdom; ^12^ Joint Research Centre for Computational Biomedicine (JRC-COMBINE), RWTH Aachen University, Faculty of Medicine, Aachen, Germany; ^13^ GROW School of Oncology and Developmental Biology and Department of Pathology, Maastricht University Medical Center, Maastricht, The Netherlands; ^14^ Max-Delbrück-Center for Molecular Medicine, Berlin, Germany; ^15^ Molecular Tumor Biology, Department of General, Visceral and Transplantation Surgery, RWTH University Hospital, Aachen, Germany

**Keywords:** cancer therapy, Annexin A1, cancer metabolism, HIF-1, induced essentiality

## Abstract

Despite the approval of numerous molecular targeted drugs, long-term antiproliferative efficacy is rarely achieved and therapy resistance remains a central obstacle of cancer care. Combined inhibition of multiple cancer-driving pathways promises to improve antiproliferative efficacy. HIF-1 is a driver of gastric cancer and considered to be an attractive target for therapy. We noted that gastric cancer cells are able to functionally compensate the stable loss of HIF-1α. Via transcriptomics we identified a group of upregulated genes in HIF-1α-deficient cells and hypothesized that these genes confer survival upon HIF-1α loss. Strikingly, simultaneous knock-down of HIF-1α and Annexin A1 (ANXA1), one of the identified genes, resulted in complete cessation of proliferation. Using stable isotope-resolved metabolomics, oxidative and reductive glutamine metabolism was found to be significantly impaired in HIF-1α/ANXA1-deficient cells, potentially explaining the proliferation defect. In summary, we present a conceptually novel application of stable gene inactivation enabling in-depth deconstruction of resistance mechanisms. In theory, this experimental approach is applicable to any cancer-driving gene or pathway and promises to identify various new targets for combination therapies.

## INTRODUCTION

Ever since the advent of the post-genomic era, our understanding of the molecular pathogenesis of cancer has increased tremendously. Modern technology has enabled accurate and detailed analyses of the molecular mechanisms and signalling networks that govern basic malignant traits such as proliferation, metastasis and therapy resistance. Clinical translation of these analyses resulted in the approval of a plethora of molecular-targeted drugs in the last decade. Unfortunately, the general efficacy of molecular-targeted drugs could not fulfill the enormous expectations that accompanied their approval [1]. We propose, as others have done before us, that the emergence of resistant tumor cell subpopulations during molecular-targeted therapy results in therapy failure and, ultimately, disease progression [1–4]. In order to develop agents for combinatorial or sequential targeted therapy it is of pivotal importance to characterize underlying resistance-mediating mechanisms [5]. Here, we introduce an experimental approach how to exploit the functional inactivation of an oncoprotein, in this case the hypoxia-inducible factor-1 (HIF-1), to identify molecules and pathways that govern resistance and would hence qualify as targets for an effective combination therapy.

The transcription factor HIF-1 was initially characterized as an important mediator of cellular adaptation to hypoxia [6]. Reduced oxygen levels impose vigorous metabolic demands on cells and HIF-1 is an essential regulator of the metabolic changes that occur in response to hypoxia [7, 8]. Of note, biologically relevant stabilization of HIF-1 under normoxic conditions can be achieved in different settings and has led to the recognition of hypoxia-independent functions of HIF-1 [7, 9]. The observation that HIF-1 is expressed by a vast number of different human cancers and their metastases, the anti-proliferative effect of HIF-1 inhibition on tumor growth *in vitro* and *in vivo*, and the pivotal importance of hypoxia/HIF-1 for chemo- and radioresistance have led to the appreciation of HIF-1 as an attractive target for cancer therapy [7, 10–12]. As outlined above, application of targeted drugs is accompanied by rapid development of therapy resistance. Against this background, we hypothesized that HIF-1 inhibitors will also be challenged by drug resistance and, subsequently, loss of clinical efficacy [7]. This is supported by the fact that cells harbouring a stable, shRNA-mediated inactivation of HIF-1α, the oxygen-regulated subunit of HIF-1, often do not show a significant growth defect *in vitro* compared to wildtype cells [13–15]. We and others have previously reported the crucial dependence of human gastric cancer progression on the functional integrity of HIF-1 [15–19]. Hence, gastric cancer would be a reasonable entity for HIF-1 inhibitors and we decided to use established human gastric cancer cell lines to perform an in-depth analysis of the functional consequences of HIF-1α inhibition.

## RESULTS

### Functional compensation of stable HIF-1α inhibition

**Figure 1:**
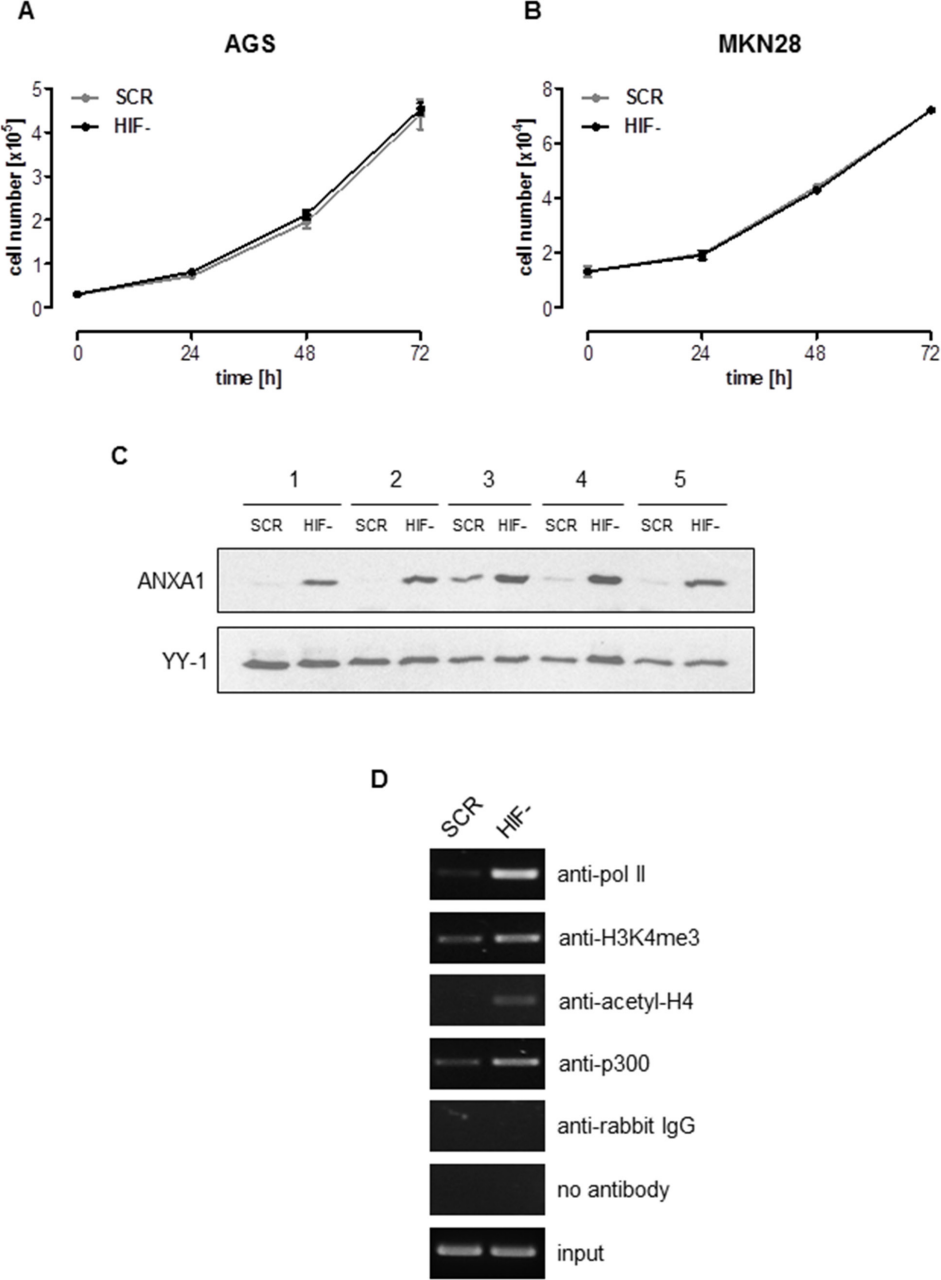
ANXA1 is a crucial mediator of HIF-1α-independent cellular proliferation (**A, B**) Anchorage-dependent proliferation of HIF-1α-deficient (HIF-) and control (SCR) AGS and MKN28 cells under normoxia. Results shown are representative of three independent experiments and values represent the mean ± SEM of triplicate determinations. (**C**) Analysis of ANXA1 expression in AGS HIF- and SCR cells by immunoblot using whole cell lysates harvested at five different time points. YY-1 served as loading control. (**D**) Chromatin status of the *ANXA1* promotor in AGS HIF- and SCR cells was determined by chromatin immunoprecipitation. Representative gels of four independent experiments are shown.

We have previously reported a highly efficient functional inactivation of HIF-1α in the gastric cancer cell lines AGS and MKN28 via lentiviral transduction of small hairpin RNAs (shRNA) (Supplementary Figure 1A and 1B) [15, 20]. Functional characterization of these cells identified HIF-1α as pivotal for the malignant phenotype of gastric cancer [15, 20, 21]. Against this background we were intrigued to note that anchorage-dependent proliferation of AGS and MKN28 cells was unaffected by stable loss of HIF-1α (Figure 1A and 1B). Moreover, growth of HIF-1α-deficient (HIF-) MKN28 xenografts was not significantly different from HIF-1α-proficient cells (scrambled control (SCR)) (Supplementary Figure 1C). HIF-2α has been shown to compensate for the loss of HIF-1α [22], leading us to analyze HIF-2α protein stabilization via immunoblot. As no effect was detectable under normoxia and only a slight increase of HIF-2α protein expression was detectable under hypoxia, we concluded that HIF-2α was not centrally involved in our experimental setup (Supplementary Figure 1D). To better understand the molecular consequences of the loss of HIF-1α we characterized the cellular transcriptome. Reassuringly, the results confirmed *HIF1A* as one of the most efficiently down-regulated genes in AGS HIF-compared to AGS SCR cells (Supplementary Figure 2A). Interestingly, a large group of genes were found up-regulated in AGS HIF-cells when compared to SCR cells (Supplementary Figure 2B). Given the central importance of HIF-1 for the malignant phenotype of AGS cells, the stable knock-down of HIF-1α can be expected to exert significant pressure on the cells to compensate for the loss of HIF-1α. We hypothesized that some of the genes that were found up-regulated in HIF-cells are mediating this compensation. To prove this hypothesis we further analyzed *Annexin A1* (*ANXA1*), one of the genes significantly up-regulated in HIF-cells (Supplementary Figure 2B). This choice was primarily based on our previous work showing reduced NF-kB activity in AGS HIF-cells [21]. ANXA1 is a known inhibitor of NF-kB and might therefore explain reduced NF-kB activity upon HIF-1α loss [23]. We first confirmed enhanced ANXA1 expression via immunoblot (Figure 1C) and quantitative real-time PCR (qPCR, Supplementary Figure 3A). Immunocytochemistry revealed that the majority of ANXA1 localized to the nucleus of HIF-1α-deficient AGS cells (Supplementary Figure 3B). Annexin A1 (ANXA1) belongs to the annexin superfamily of calcium and phospholipid binding proteins [24]. While ANXA1 expression is inducible by hypoxia in a HIF-1-dependent fashion, a functional role for ANXA1 under conditions of HIF-1 depletion has not been reported thus far [25]. Via chromatin immunoprecipitation (ChIP) we analyzed activating chromatin marks, pan-acetylation of histone H4 and tri-methylation of lysine 4 of histone H3 (H3K4) at the *ANXA1* promoter of AGS SCR and HIF-cells. ChIP revealed an increase in pan-acetylation of histone H4 and tri-methylation of H3K4 in the promoter region of HIF-cells compared to SCR cells (Figure 1D). In addition, binding of the histone acetyltransferase p300 to the *ANXA1* promoter was observed, suggesting p300 being responsible for increased acetylation. Increased transcriptional activity of the *ANXA1* promoter was further indicated by recruitment of RNA polymerase II (Figure 1D). These data argue for an involvement of epigenetic mechanisms for the regulation of *ANXA1* transcription upon HIF-1α loss.

### Simultaneous knock-down of HIF-1α and ANXA1 resembles induced essentiality

**Figure 2:**
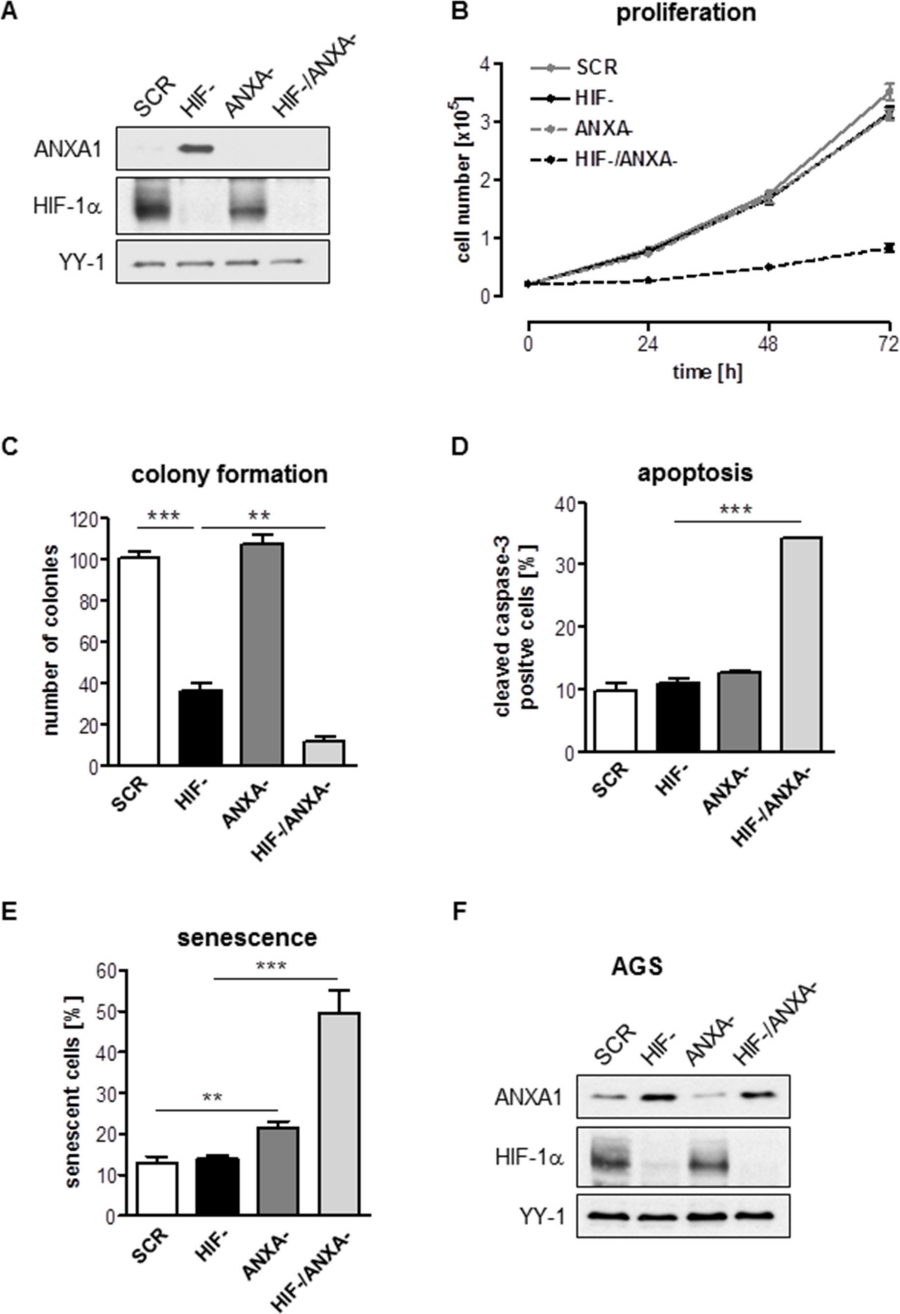
Effects of combined inhibition of HIF-1α and ANXA1 on AGS cell proliferation (**A**) Representative immunoblot analysis of ANXA1 and HIF-1α in control (SCR), HIF-1α-deficient (HIF-), ANXA1-deficient (ANXA-) and double knock-down (HIF-/ANXA-) cells. YY-1 served as loading control. (**B, C**) Anchorage-dependent (B) and anchorage-independent (C) proliferation. Results shown are representative of three independent experiments and values represent the mean ± SEM of triplicate determinations (**P < 0.01; ***P < 0.001). (**D**) Apoptosis was assayed by cleavage of caspase-3 followed by FACS analysis. Values are means ± SEM (***P < 0.001). (**E**) Senescence was quantified 96 h after cultivation by measurement of SA-β-Gal activity. Values are means ± SEM (**P < 0.01; ***P < 0.001). (**F**) Representative immunoblot analysis of ANXA1 and HIF-1α. Cells were passaged regularly and grown as monolayer cultures under recommended culture conditions for four weeks. YY-1 served as loading control.

To explore the cellular response upon combined inhibition of HIF-1α and ANXA1, we established a stable inhibition of ANXA1 in HIF- and SCR cells via lentiviral RNAi. Efficacy of the ANXA1 knock-down (KD) was shown by immunoblot (Figure 2A). Strikingly, simultaneous KD of HIF-1α and ANXA1 (HIF-/ANXA-) resulted in near complete cessation of both anchorage-dependent and –independent proliferation while inhibition of ANXA1 alone remained without effect in AGS cells (Figure 2B and 2C, Supplementary Figure 4A–4D). While inhibition of HIF-1α or ANXA1 alone did not result in relevant apoptosis, combined inhibition strongly enhanced both pre-G1 fraction (Supplementary Figure 5A–5D) and caspase-3 activation (Figure 2D). In addition, robust induction of senescence, an important failsafe mechanism, was detected in HIF-/ANXA-cells (Figure 2E). We were intrigued by the observation of some degree of proliferation in the HIF-/ANXA-cells and hypothesized that the proliferating cell fraction had escaped the double knock-down. Indeed, immunoblot analysis of double KD cells after four weeks in culture displayed a clear ANXA1 protein signal, revealing that “true” double KD cells are not able to survive and arguing that AGS cells cannot compensate the simultaneous inactivation of HIF-1α and ANXA1 (Figure 2F). Next, we sought to confirm these results in a second gastric cancer cell line. As opposed to AGS, MKN28 cells displayed strong ANXA1 protein expression already under control conditions and the loss of HIF-1α did not affect ANXA1 protein levels (Supplementary Figure 6). Interestingly, inactivation of ANXA1 resulted in near complete death of MKN28 cells. Only very few cells were able to survive the lentiviral transduction and, when analyzed after a recovery period of 4 weeks, these cells turned out to be ANXA1-positive (Supplementary Figure 6). These results point towards a crucial and hitherto unrecognized role of ANXA1 for survival and proliferation of MKN28 cells.

### Reductive carboxylation is active in AGS wild type cells under normal cell culture conditions

**Figure 3:**
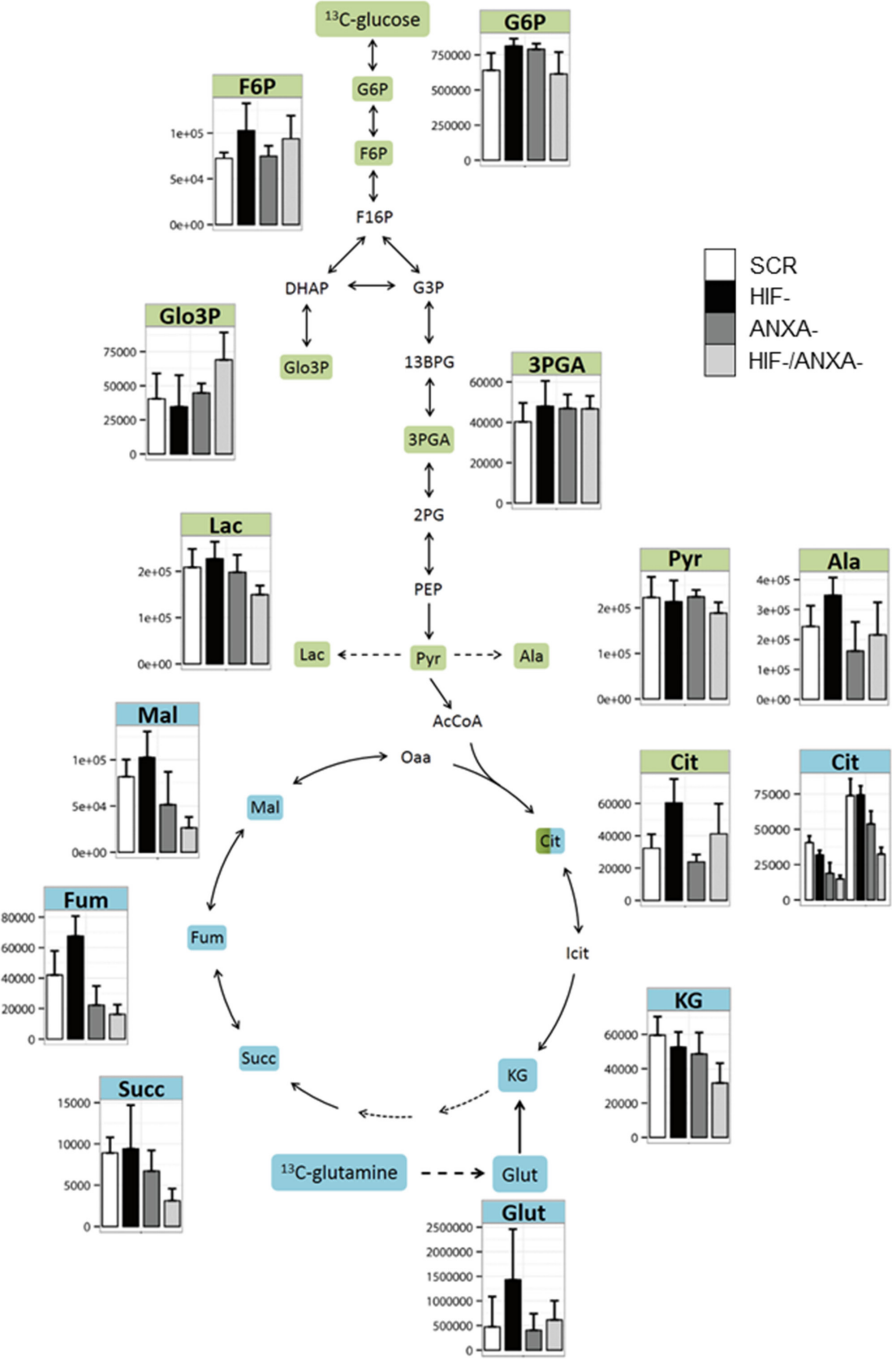
Activity of the central carbon metabolism of AGS cells estimated by pSIRM Glycolytic and TCA cycle activity of the different AGS knock-down derivatives was determined by ^13^C-glucose (green) and ^13^C-glutamine (blue) pSIRM experiments (*n* = 3). Labelled quantities of the different intermediate metabolites are shown. Abbreviations: 13BG: 1, 3 bis-phosphoglycerate; 2PG: 2-phosphoglycerate; 3PG: 3-phosphoglycerate; AcCoA: acetyl coenzyme A; Ala: Alanine; Cit: citrate; DHAP: dihydroxyacetonate-phosphate; F6P: fructose 6-phosphate; F16P: fructose 1, 6 bis-phosphate; Fum: fumarate; G3P: glyceraldehyde-3-phosphate; G6P: glucose 6-phosphate; Glut: glutamate; Icit: iso-citrate; KG: alpha-ketoglutarate; Lac: lactate; Mal: malate; Oaa: oxaloacetate; PEP: phosphoenolpyruvate; Pyr: pyruvate; Succ: succinate.

**Figure 4:**
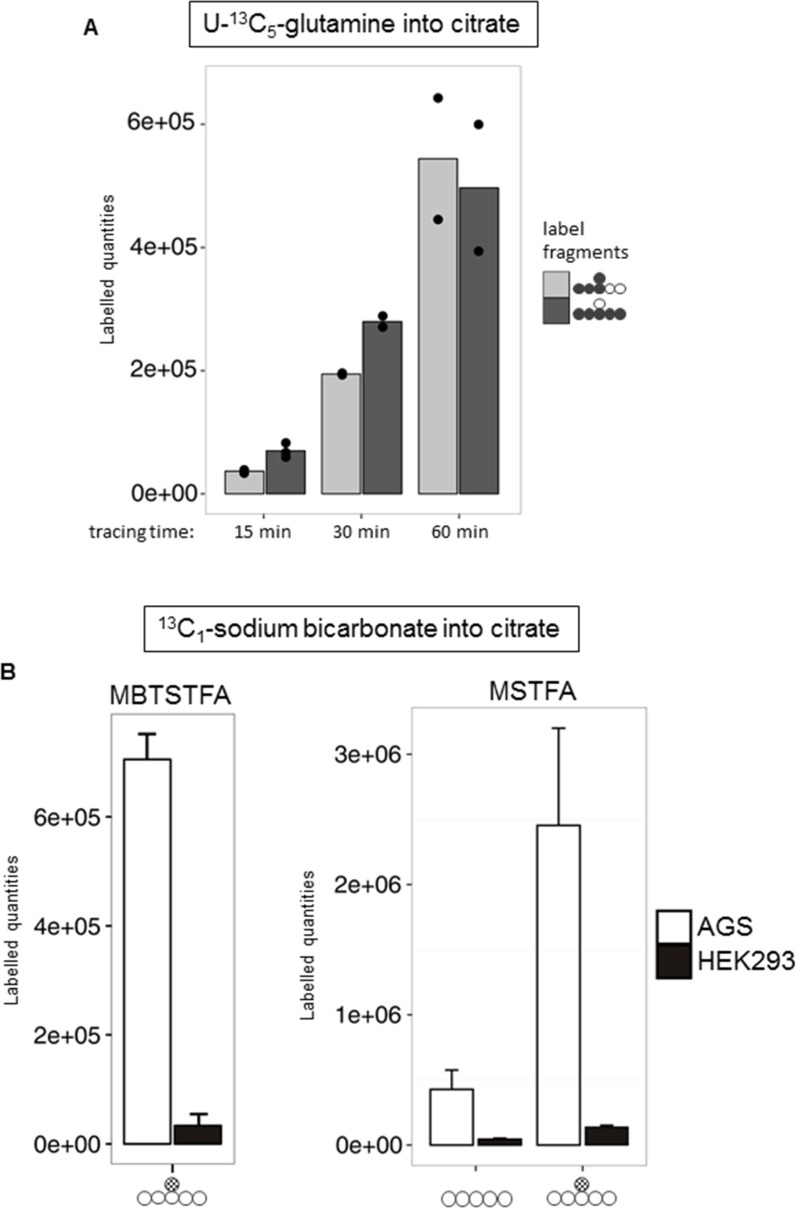
Evidence for reductive TCA metabolism via ^13^C_5_-glutamine and ^13^C_1_-HCO_3_ labelling (**A**) Time series pSIRM analysis of AGS wild type cells with U-^13^C_5_-glutamine for 15, 30 and 60 min (*n* = 3, *n* = 2 and *n* = 2, respectively) under normoxic conditions. Labelled quantities for citrate (looking at m + 3 and m + 5 mass shift of the C5 fragment; for easier recognition full metabolite is depicted by circles) at these three time points are shown (mean ± SD, *n* = 3). (**B**) Comparison of the derivatization agents MBTSTFA and MSTFA for citrate detection. Left (MBTSTFA): AGS wild type and HEK293 cells were labelled with ^13^C_1_-sodium bicarbonate for 20 min under normoxic conditions. Carbon fixation was observed by a mass shift on m + 1 of citrate (m/z = 459). Right (MSTFA): Same labelling conditions, measurement in splitless mode for improved detection of the C6-fragment. Depicted is the C5-fragment (m/z = 273) compared to the m + 1 of the C6-fragment (m/z = 375) of citrate. Values are means ± SD of three independent experiments.

**Figure 5:**
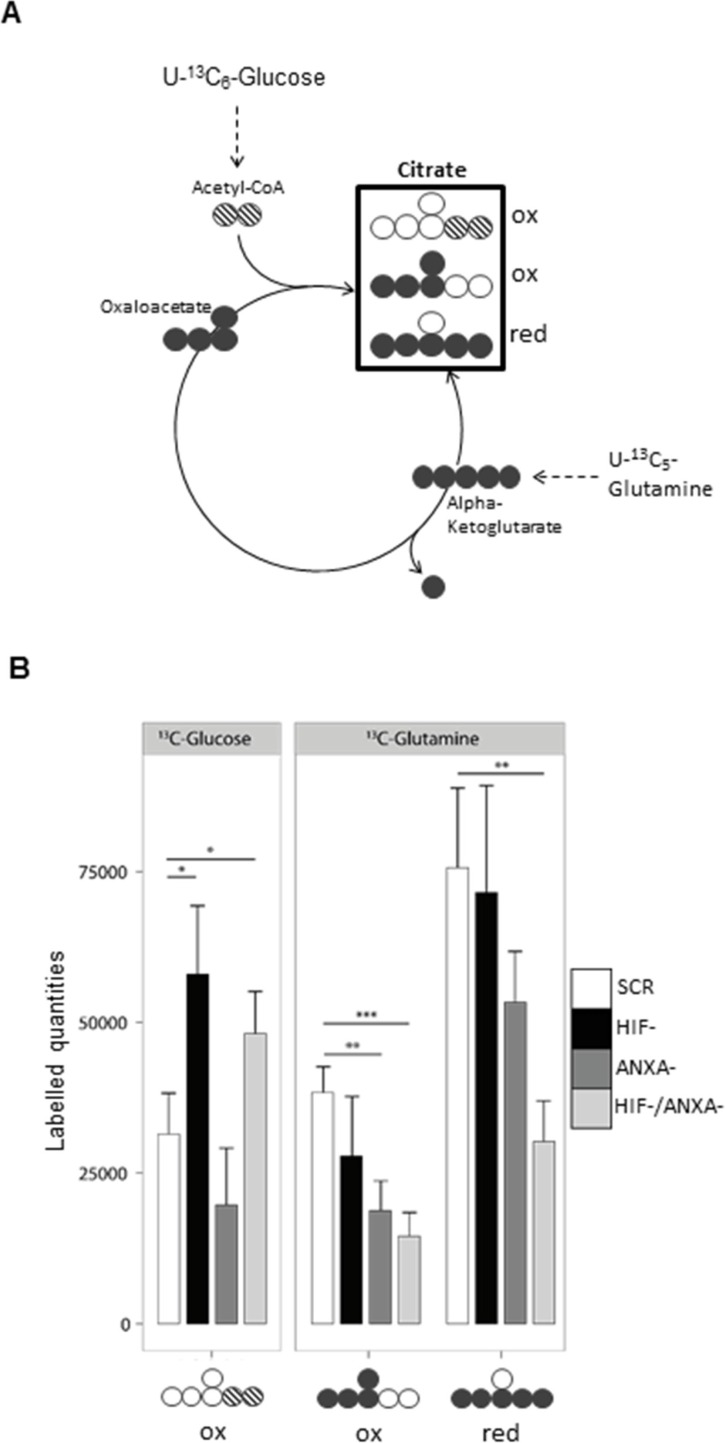
Reductive carboxylation is reduced in HIF-/ANXA-AGS cells (**A**) Schematic representation of the different mass isotopomers of citrate and the corresponding origin via the different labelling substrates U-^13^C_6_-glucose and U-^13^C_5_-glutamine. Three different mass isotopomers of citrate are the result of oxidative (ox) and reductive (red) carboxylation (
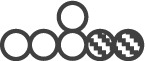
, 
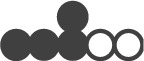
 and 
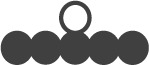
). (**B**) Shown are the corresponding labelled quantities of the detectable C5-fragment of citrate (*n* = 3; mean ± SD; *P < 0.05; **P < 0.01; ***P < 0.001)) for control (scr) and the different knock-down AGS cells.

To better understand the molecular nature of the interaction between HIF-1α and ANXA1 in AGS cells, we employed pulsed stable isotope-resolved metabolomics (pSIRM) [26]. This method allows for dynamic measurements of the central carbon metabolism (CCM), the ultimate source of energy and building blocks essential for cell growth. We analyzed carbon routing and pathway activity within the CCM of AGS cells using U-^13^C_6_-glucose and U-^13^C_5_-glutamine, respectively (schematically outlined in Supplementary Figure 7). Incorporation of ^13^C-carbon atoms in downstream metabolites following glycolysis (e.g. pyruvate and lactate) and the TCA cycle (e.g. citrate) was monitored by GC-MS (Figures 3–5). We found isotopically labelled glutamate, α-ketoglutarate, succinate and further di-carboxylic acids towards citrate as the expected result of oxidative TCA cycle after labelling with U-^13^C_5_-glutamine (Figure 3). Thereafter, we focused on citrate as this metabolite is the linchpin of glycolysis, TCA cycle and fatty acid synthesis [27]. We detected citrate isotopically labelled with five ^13^C-carbons (Figure 4A). This pattern cannot be explained by oxidative TCA cycle metabolism as oxaloacetate cannot provide more than four carbon atoms (Supplementary Figure 7B). It may be a result of the reductive activity of isocitrate dehydrogenase (IDH), promoting reductive TCA cycle metabolism (Supplementary Figure 7C) [28–31]. The increase of labelled citrate quantities over time supports the idea that both oxidative and reductive carboxylation take place simultaneously in AGS cells. To analyze whether direct carboxylation or other pathways contribute to the observed mass isotopomers in citrate, we isotopically labelled AGS cells with^13^C_1_-carbon dioxide (4B, Supplementary Figure 7D). Instead of using the gaseous form, buffer-free media was supplemented with ^13^C_1_ sodium bicarbonate. Besides the standard derivatization for GC-MS (with MSTFA), the derivatization agent MBTSTFA was used to allow for detection of the full C6 fragment of citrate [32]. This enabled determination of the positional ^13^C carbon incorporation into citrate (Figure 4B). The mass fragment 273 m/z of citrate (resulting from the MSTFA derivatization) contains five carbon atoms. This fragment revealed only minor label incorporation after ^13^C_1_ sodium bicarbonate tracer experiments (Figure 4B, right panel). However, the 375 m/z fragment, representing the full metabolite after MSTFA derivatization, revealed one labelled carbon (Figure 4B, right panel). This was confirmed via MBTSTFA derivatization and analysis of the full metabolite fragment of 459 m/z (Figure 4B, left panel). AGS cells incorporated a much higher yield of ^13^C_1_-sodium bicarbonate into citrate than HEK293 cells, supporting the concept of direct carbon fixation in the TCA cycle in these cells. Our results strongly suggest the direct incorporation of CO_2_ into citrate via carboxylation of α-ketoglutarate and thus the presence of a reductive TCA metabolism in AGS cells under normoxic conditions. A carboxylation of pyruvate (to form oxaloacetate by pyruvate carboxylase) would result in ^13^C incorporation into citrate at a different position after fusion with acetyl-CoA (Supplementary Figure 9).

### HIF-1α and ANXA1 synergistically influence glutamine metabolism of AGS cells

To deconstruct the molecular nature of the observed synthetic lethality between HIF-1α and ANXA1, we analyzed the corresponding knock-down cells with pSIRM (Figures 3 and 5). Using the labelled quantities of lactate as a read-out for aerobic glycolysis, HIF-/ANXA-cells showed decreased glycolytic activity (Figure 3). In contrast, the labelled quantities of pyruvate suggested a similar overall performance of glycolysis in all knock-down cells. HIF-1α loss resulted in a higher TCA cycle activity judging from the labelled quantities in citrate, indicating shifted carbon routing from pyruvate downstream towards mitochondrial metabolism (Figure 5B). HIF-1 is known to repress pyruvate shuttling into the TCA cycle via its target gene pyruvate dehydrogenase kinase [33]. Knock-down of ANXA1 led to decreased citrate formation via acetyl-CoA as judged by the labelled quantities of citrate resulting from U-^13^C_6_-glucose (Figure 5B). In addition, the activity of glutamine metabolism decreased upon ANXA1 inactivation judging the label incorporation from U-^13^C_5_-glutamine (Figure 5B). Strikingly, the simultaneous knock-down of both HIF-1α and ANXA1 revealed significantly stronger effects as both canonical and reductive TCA metabolism were impaired, arguing for a decrease of TCA cycle activity and less efficient glutaminolysis.

### Loss of MYC underlies the proliferation defect of HIF-1α/ANXA1-deficient cells

**Figure 6:**
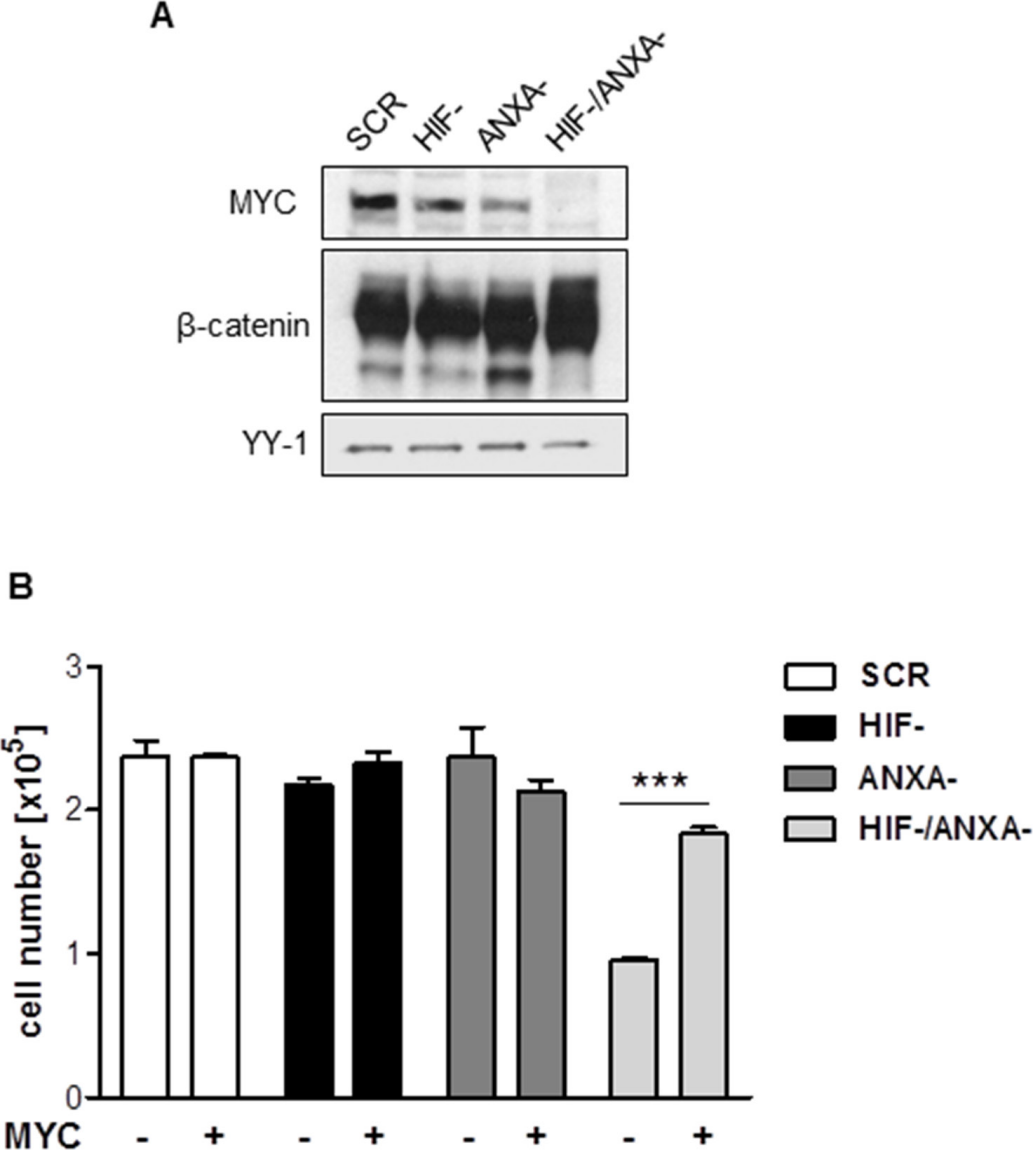
Contribution of MYC to the growth defect in HIF-1α/ANXA1-deficient cells (**A**) Representative immunoblot analysis of MYC and β-catenin in control (SCR), HIF-1α-deficient (HIF-), ANXA1-deficient (ANXA-) and double knock-down (HIF-/ANXA-) AGS cells. YY-1 served as loading control. (**B**) Re-expression of *MYC* was achieved by transduction with retroviruses containing human *MYC* and anchorage-dependent proliferation was analyzed 14 days post transduction. Results shown are representative of three independent experiments and values represent the mean ± SEM of triplicate determinations (***P < 0.001).

Glutamine metabolism and citrate homeostasis in cancer cells are centrally regulated by the oncogene *MYC* [34, 35]. MYC has been described as a central driving force of the malignancy of AGS gastric cancer cells [36]. Strikingly, simultaneous inhibition of HIF-1α and ANXA1 resulted in complete loss of MYC protein expression (Figure 6A). To analyse the functional importance of the loss of MYC for the defective proliferation and survival of HIF-1α/ANXA1-deficient cells, we re-introduced MYC via retroviral transduction (Supplementary Figure 10). As shown in Figure 6B, re-expression of MYC in HIF-/ANXA-cells resulted in a near complete rescue of proliferation, strongly supporting the functional involvement of MYC in the observed growth defect. As MYC represents a β-catenin target gene, we investigated a potential causal role of this pathway. As can be seen in Figure 6A, protein expression of β-catenin remained unchanged in all the cellular derivatives, arguing against a functional importance of Wnt/β-catenin in this setting.

### Comparative analysis of *HIF1A, ANXA1* and *MYC* expression in gastric cancer cell lines and primary tissues

**Figure 7:**
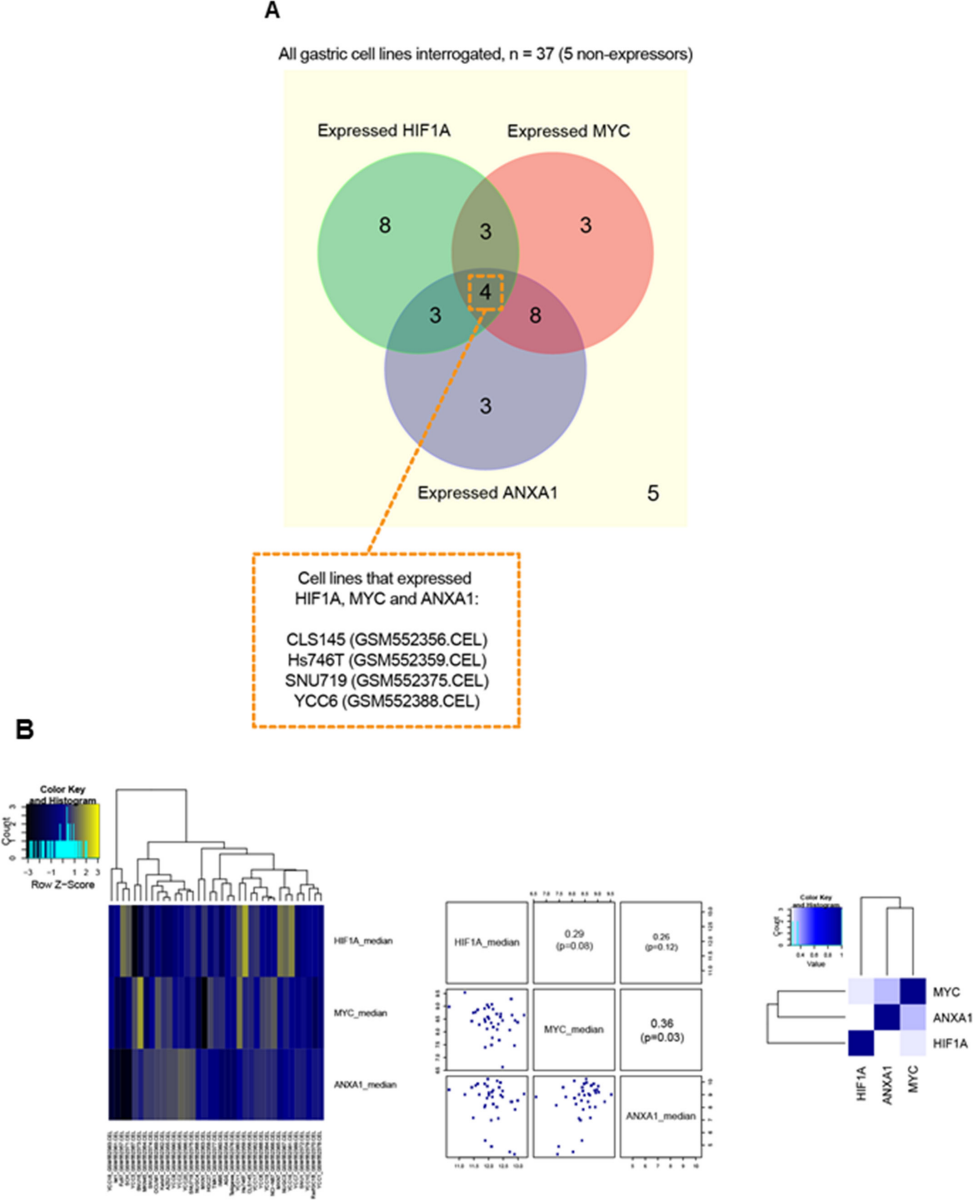
Comparative analysis of HIF1A, ANXA1 and MYC expression in gastric cancer cell lines (**A**) A Venn diagram to illustrate the cell line samples with overlap of expression for HIF1A and/or ANXA1 and/or MYC. As shown, four cell lines, namely CLS145, Hs746T, SNU719 and YCC6, expressed all 3 genes (i.e. HIF1A, ANXA1 and MYC). Generally, HIF1A and/or ANXA1 expressors show expression mutual exclusivity. (**B**) Heatmap (left) shows results of hierarchical clustering of HIF1A, MYC and ANXA1 gene expression across the 37 cell line samples. In general, apart from co-expression pattern seen for MYC and ANXA1, most of the gene-pairs appeared to have patterns of expression mutual exclusivity. A pairs-plot (middle) and a correlation-matrix heatmap (right) further support these findings i.e. a significant correlation (*p* < 0.05) for co-expression was found only for MYC and ANXA1.

To test the reproducibility of the above outlined data, we evaluated the gene expression patterns of *HIF1A*, *ANXA1* and *MYC* in other gastric cancer cell lines (*n* = 37 [37]). Of the 37 cell lines, 29 expressed *HIF1A* and/or *ANXA1* (Figure 7A). Among these *HIF1A* and/or *ANXA1* expressors, the majority (22/29 = 75.9%) expressed *HIF1A* and *ANXA1* in a mutual exclusive manner. Additionally, assessment of these cell lines revealed a significant co-expression pattern for *MYC* and *ANXA1* (Figure 7B). To investigate the potential interaction between HIF-1α, ANXA1 and MYC in primary gastric cancer, we performed a bioinformatics analysis of co-occurrence and mutual exclusivity using a RNA-seq data-set from the TCGA data-base with 265 samples of stomach adenocarcinoma (cBioPortal [38, 39]). We considered that genes with an expression higher than one standard deviation above the mean are highly expressed on the respective sample and performed a pairwise hyper-geometric test to assess the co-occurrence and mutual exclusivity probability. This analysis revealed a tendency for mutual exclusivity between *HIF1A* and *ANXA1* gene expression (odds ratio between 0 and 0.1), however, statistical significance was not reached (*p*-value 0.783). Moreover, *ANXA1* and *MYC* tended to co-occur in the same stomach adenocarcinoma samples at the gene expression level (odds ratio between 2 and 10). Finally, we performed immunohistochemistry for HIF-1α and ANXA1 on a tissue microarray comprising 397 samples of human gastric adenocarcinoma. Given the function of HIF-1 as a transcription factor, only nuclear signals were quantified and analyzed, while ANXA1 protein has been described to exert functions at various cellular locations, e.g. cell membrane, cytoplasm and nucleus (Supplementary Figure 11). Interestingly, 80.4% of HIF-1α-positive gastric cancers were negative for ANXA1, further supporting the notion of mutual exclusivity. Taken together, the comparative analysis of HIF-1α, ANXA1 and MYC expression in human gastric cancer samples confirmed the *in vitro* data, further indicating a potential functional association between HIF-1α, ANXA1 and MYC in this tumor entity.

## DISCUSSION

Drug resistance is a pivotal obstacle in clinical cancer care. While a plethora of molecular-targeted drugs was newly approved for cancer therapy in recent years, this did not translate into improved overall survival for the majority of cancer entities. It is widely assumed that secondary resistance underlies the suboptimal efficacy of most targeted therapeutics and that combined inhibition of cancer-driving pathways represents an attractive approach to circumvent the emergence of drug resistance [2, 3]. Identification of pathways and molecules that qualify as targets for an effective combination therapy is therefore of paramount importance. Our data support the notion that the stable inhibition of a given cancer-driving factor in an established cancer cell line can serve as a valuable tool to identify targets for effective combinatorial approaches.

HIF-1 was originally described as the principle mediator of cellular adaptation to hypoxia [6]. The majority of cancer cell lines and experimental tumor models depends on HIF-1 for growth and systemic spread, resulting in the general appreciation of HIF-1 as an oncoprotein and an attractive target for cancer therapy [40]. Given the rapid emergence of resistance against molecular-targeted drugs, we hypothesized that drug resistance will also occur during treatment with HIF-1 inhibiting agents and sought to characterize the underlying molecular mechanisms. To achieve this, we took advantage of a previously established shRNA-based stable inhibition of HIF-1α in a number of cancer cell lines [15, 20]. Strikingly, stable inhibition of HIF-1α did not interfere with cellular proliferation in the majority of analyzed cell lines. This rather unexpected observation could either mean that HIF-1 is not essential for malignant growth of gastric cancer cells or that the cells were able to compensate the stable loss of HIF-1α over time, enabling them to thrive in a HIF-1-independent fashion. Via transcriptome analyses we were able to identify a group of genes with enhanced expression upon HIF-1α loss. Repression of target gene activity by HIF-1 has been reported and can occur via binding of HIF-1 to a so-called “reverse HRE” [41, 42]. Hence, enhanced gene expression in HIF-1α-deficient cells could be explained by active repression of these genes when HIF-1 is present. We hypothesized, however, that some of the genes with enhanced expression might not be actively repressed in WT cells, but rather get activated as part of the cellular adaptation process to compensate for the loss of HIF-1α [43]. These genes would therefore be crucial for HIF-1-independent survival and could represent interesting targets for a combination therapy with HIF-1 inhibitors. To support our hypothesis we analysed the role of annexin A1 (ANXA1) for growth of HIF-1α-deficient cells. ANXA1 is a member of the annexin protein family, named after their ability to annex to phospholipids in the cell membrane [44]. ANXA1 is best known for its anti-inflammatory role and is considered to be a therapy target for chronic inflammatory conditions [45]. We chose ANXA1 for further analysis due to its suppressive action on the NF-kB pathway [23]. As reported previously by us, the NF-kB pathway is strongly repressed in gastric cancer cells upon stable loss of HIF-1α [21]. Studies based on cDNA microarray analyses reported differentiation-dependent expression of ANXA1 in human gastric cancer with higher expression in diffuse-type than in intestinal-type gastric cancers [46, 47]. Immunohistochemistry data obtained with tissue microarrays reported loss of ANXA1 in 64% of primary gastric cancers and 86% of lymph node metastases, arguing for a tumor-suppressive function of ANXA1 in gastric cancer [48]. Our data point towards a crucial and hitherto unreported role of ANXA1: Functional compensation of stable HIF-1α loss (Supplementary Figure 12). Numerous international research groups, including ourselves, have published data pointing towards a crucial pro-tumorigenic role of HIF-1 in gastric cancer [15, 21, 49]. Our data demonstrate that gastric cancer cells are able to functionally compensate the loss of HIF-1α, arguing for the emergence of resistance during a potential therapy of gastric cancer with HIF-1-inhibiting agents. We could show that simultaneous inhibition of HIF-1α and ANXA1 leads to robust and non compensable cell death, arguing for a combination therapy of HIF-1 and ANXA1 inhibitors for gastric cancer. In this setting, the anti-inflammatory function of ANXA1 needs to be regarded: ANXA1 inhibiting substances might result in enhanced inflammatory activity, potentially causing undesirable side effects. One possible strategy to avoid this scenario is to target the nuclear function of ANXA1. As outlined above, the enhanced ANXA1 protein in HIF-1α-deficient cells is almost exclusively found in the nucleus. The nuclear function of ANXA1 differs from cell membrane-bound ANXA1, the latter being mediated by binding to its specific membrane receptor ALXR [44], the former by direct binding to DNA. Hence, specific inhibitors of nuclear ANXA1 activity would be ideal combination partners for pharmaceutical HIF-1 inhibitors without the risk of enhanced inflammation.

As HIF-1 is known to regulate various metabolic enzymes, we explored the nature of defective proliferation in HIF-1α/ANXA1-deficient cells using pulsed stable isotope-resolved metabolomics (pSIRM). We applied this technology as a diagnostic tool and analysed the dynamics of glycolysis and glutaminolysis using ^13^C-labelled substrates at in-stationary isotope incorporation states. This approach led to the observation that both oxidative and reductive carboxylation (RC) take place simultaneously in AGS wildtype cells. The presence of RC was rather unexpected as our experiments were performed under normoxic conditions and RC is usually favoured under conditions of hypoxia [50]. Thus far, normoxic RC has only been reported in cells with a mutated von Hippel-Lindau tumor suppressor gene or defective mitochondria [30, 31]. Of note, HIF-1 was identified as the main regulator of RC under both hypoxic and VHL-defective conditions [51, 52]. Against this background it is important to note that AGS cells display functional HIF-1 under normoxic conditions, a phenomenon seen in various cancer cell lines and mainly attributable to activation of oncogenes (e.g. *ras*), inactivation of tumor suppressor genes (e.g. *vhl*), reactive oxygen species or metabolite-mediated PHD inhibition [12]. Hence, normoxic RC in AGS cells could principally be mediated by HIF-1 activity. However, as stable inactivation of HIF-1*a* in AGS cells did not result in significant reduction of RC, HIF-1 seems dispensable in this setting and the regulatory nature of RC in these cells remains elusive.

Therapy resistance – either primary or secondary- results in therapy failure, which in the clinical setting almost always means cancer progression and decease of the affected patient. It is therefore not surprising that therapy resistance and the governing molecular mechanisms are usually discussed as phenomena that need to be either avoided or tackled. While this is certainly true and the efforts to better characterize the molecular nature of therapy resistance are undeniably justified, our data point towards the appreciation of therapy resistance as a door opener to innovative and, eventually, more efficient forms of cancer therapy. The adaptation processes that enable the survival of neoplastic cells in response to antiproliferative therapies result in usage of alternative pathways to maintain the malignant phenotype. We propose to exploit the molecular mechanisms of this adaptation to identify new therapy targets. Our results demonstrate the applicability of this approach, starting with the analysis of cell lines and validating the results with RNA and protein expression data from a large collection of human cancer samples.

Taken together, we believe that stable HIF-1α-deficient tumor cells represent precious experimental tools as they potentially mirror molecular events that might confer resistance of human tumors towards HIF-1 inhibitors. Our work applying “omics-“analyses to such cells showed robust activation of factors centrally involved in the regulation of apoptosis, survival, cell cycle and redox balance upon inactivation of HIF-1α [43]. We believe that this experimental approach will lead to the identification of multiple pathways whose inhibition holds the potential to enhance the antiproliferative efficacy of HIF-1 inhibitors. In addition, this strategy should prove valuable to deconstruct resistance to other cancer-driving factors, e.g. activated oncogenes and tyrosine kinases, as the basic methodology is applicable to any target of choice.

## MATERIALS AND METHODS

### Ethics statement

Investigation has been conducted in accordance with the ethical standards and according to the Declaration of Helsinki and according to national and international guidelines and has been approved by the authors’ institutional review board.

### Cell lines and culture conditions

The human gastric cancer cell line AGS (CRL-1739, ATCC, Rockville, USA) and MKN28 (Riken, Ibaraki, Japan) were grown as monolayer cultures in recommended medium. Generation of AGS and MKN28 cells stably expressing either shRNA specifically targeting HIF-1α (HIF-) or unspecific control shRNA (scrambled, “SCR”) was published previously [15, 20]. To stably knock-down annexin A1 (ANXA1), AGS cells were transduced with two lentiviruses containing different shRNA sequences against human ANXA1 (MISSION^®^ shRNA lentiviral transduction particles; Sigma-Aldrich, Deisenhofen, Germany). AGS cells stably re-expressing *MY*C were generated by double transduction with retroviruses containing human *MYC* [53].

### Cell growth assay

For determination of anchorage-dependent cell growth, 3 × 10^4^ cells were seeded in triplicate into 24-well plates. Cells were trypsinized and counted every 24 h using a hemocytometer. Media were not changed for the duration of the experiment. Anchorage-independent growth was assessed by human tumor clonogenic assay as described before [54].

### Tumor xenograft growth

Female NOD/SCID mice (*n* = 3) purchased from Taconic (Denmark) were inoculated at day 0 with 10^7^ cells of the MKN28 HIF- or SCR cells. Tumor growth was monitored twice per week by caliper measurements and calculation of tumor volumes (TV). The animal experiments were approved by the local ethical committee (LaGeSo Berlin) and performed according to the “Guidelines for the welfare and use of animals in cancer research” [55].

### Determination of cell cycle distribution, proliferation and apoptosis by flow cytometry

Cell cycle distribution including the pre-G_1_ fraction was determined from DNA histograms as described before [54]. Apoptosis was quantified from detection of active, cleaved caspase-3 by flow cytometry using an Alexa Fluor^®^ 488-conjugated antibody (Cell Signaling Technology, Danvers, Massachusetts, USA) according to the manufacturer's protocol. Cell proliferation and the rate of DNA synthesis were determined by flow cytometry on AGS cells labelled with anti-BrdU antibody (AbD Serotec, Puchheim, Germany) and propidium iodide (PI, Sigma-Aldrich) according to the manufacturer's protocol. Briefly, cells were pulse labelled with 10 μM BrdU (Sigma-Aldrich) for 60 min, acid-treated and stained with an anti-BrdU and a secondary APC-conjugated antibody for determination of DNA synthesis and with 20 μg/ml PI for determination of total DNA content.

### Quantification of senescence

Senescence-associated β-galactosidase staining was performed according to the manufacturer‘s protocol (Sigma-Aldrich).

### Immunoblot analysis

Whole cell lysates or nuclear protein extracts were prepared as previously described [54, 56], then resolved on a sodium dodecyl sulfate-polyacrylamide gel, and transferred to a nitrocellulose membrane (Amersham Biosciences, Freiburg, Germany). Blots were probed with antibodies against actin (Sigma-Aldrich), annexin A1 (BD Biosciences, Heidelberg, Germany), β-catenin (BD Biosciences), MYC (Santa Cruz Biotechnology, Santa Cruz, USA), HIF-1α (Cayman Chemical, Ann Arbor, USA), HIF-2α (Novus Biologicals, Littleton, USA) and YY-1 (Santa Cruz Biotechnology). Secondary antibodies were conjugated to horseradish peroxidase (Dianova, Hamburg, Germany) and peroxidase activity was visualized using the Western Lightning™ Chemiluminescence Reagent Plus (Perkin Elmer Life Sciences, Boston, Massachusetts, USA).

### Immunocytochemical detection of annexin A1

Cells grown onto sterile coverslips were fixed in 4% paraformaldehyde for 20 min at RT, washed twice with PBS and permeabilized in 0.1% Triton X-100/PBS for 10 min. Following permeabilization, cells were washed twice with PBS, blocked for 30 min in PBS with 2% nonfat dried milk, washed twice again and were then incubated with an antibody to human annexin A1 in 0.1% BSA/PBS at RT. Cells were washed four times in PBS before incubation with a Cy3-conjugated antibody followed by two additional washes with PBS and final fixation in ethanol. Cells were mounted in Elvanol and immunofluorescence was evaluated on a confocal laser scanning microscope LSM510 (Zeiss, Jena, Germany).

### Chromatin immunoprecipitation (ChIP)

ChIP analysis was conducted in cells grown to confluency. For crosslinking, cells were incubated with 1% formaldehyde/PBS for 1 min at RT and the reaction was stopped by the addition of ice-cold 0.125 M glycin/PBS. Cells were washed twice with PBS and then rapidly collected by scraping in PBS with subsequent centrifugation. Cell pellets were resuspended in RIPA buffer [10 mM Tris (pH 7.5), 150 mM NaCl, 1% NP-40, 1% desoxycholic acid, 0.1% SDS, 1 mM EDTA] supplemented with protease inhibitors (complete EDTA-free protease inhibitor cocktail tablets; Roche) and the chromatin was sheared by sonication. Lysates were clarified by centrifugation. Immunoprecipitations were performed using anti-mRNA polymerase II (Pol II, Santa Cruz Biotechnology), anti-acetyl-Histone H4 (Merck Millipore, Billerica, USA), anti-H3K4me3 (abcam, Cambridge, United Kingdom), anti-p300 (Santa Cruz Biotechnology) and a rabbit control IgG antibody (abcam) at 4°C overnight. Immune complexes were captured by addition of 40 μl of protein A/G agarose slurry for 2 h. Following pull down the agarose beads were subsequently washed twice with RIPA buffer, followed by one wash-step each using high-salt buffer [2 M NaCl, 10 mM Tris (pH 7.5), 1% NP-40, 0.5% desoxycholic acid, 1 mM EDTA], RIPA buffer and TE buffer [10 mM Tris (pH 7.5), 1 mM EDTA]. Immune complexes were extracted by addition of 55 μl elution buffer (TE buffer containing 1% SDS) and shaking for 20 min at 1,200 rpm (30°C). The crosslinking was reversed by incubation for 30 min at 37°C in the presence of RNase (1 μg/20 μl, AppliChem), followed by proteinase K treatment (1 μg/2.05 μl, AppliChem, Darmstadt, Germany) for 6 h at 37°C and a final incubation for 6 h at 65°C. DNA was purified using the PCR purification kit (Qiagen). Purified ChIP DNA as well as input DNA was amplified by PCR using PAN Hotstart DNA Polymerase (PAN Biotech, Aidenbach, Germany). PCR products were separated by agarose gel electrophoresis and detected by ethidium bromide staining. Primer sequences are supplied in Table E1.

### Quantitative real-time PCR analysis

Total cellular RNA was isolated using the RNeasy Mini Kit according to the manufacturer's protocol (Qiagen) and first strand cDNA was synthesized with an oligo (dT) primer and a SuperScript™ First Strand Synthesis System (Invitrogen, Rockville, Maryland, USA). Quantitative real-time PCR analysis was conducted by using TaqMan PCR Universal Mastermix (for β-actin) or SYBR GREEN PCR Master Mix (for Annexin A1; Applied Biosystems, Darmstadt, Germany). Quantification was performed by the comparative ΔC_T_ method normalizing C_T_-values to β-actin.

### Global gene expression analysis

Total RNA was quantified by a Nanodrop 2000 spectrophotometer and quality checked using a total RNA 6000 nano chip on a 2100 Agilent Bioanalyzer (Agilent Technologies, Böblingen, Germany). All samples were of high quality with RNA integrity numbers (RIN) of 9.9 or 10. On a scala from 1 to 10, a RIN of 10 represents the highest RNA integrity. For array hybridization, 500 ng of RNA were used for the synthesis of biotinylated cRNA employing a linear amplification kit (Ambion, Darmstadt, Germany). The biotinylated cRNA was then hybridized on a HumanHT-12 v4 Expression BeadChip (Illumina, San Diego, USA). Hybridization, washing, detection with Cy3-strepatvidin and scanning were performed on the Illumina BeadStation 500 platform (Illumina) according to the manufacturer's instructions. Expression data were normalized using RMA and filtered using the beadarray package in the statistical language R [57]. Different array versions for the biological replicates were compared based on probe IDs common to all three independent experiments. Genes that displayed consistent expression below the 50% expression quantile were discarded. Expression data from technical replicates were averaged for further analysis. To account for the dynamic variation within the signal range of the arrays, fold changes of sample to control were z-transformed using an error model as described previously [58]. We then defined those genes as significantly regulated where the absolute *z*-value was consistently among the top 10% in each experiment, and had consistent sign of regulation. Using 1000 triple random draws with replacement, we estimated that the FDR of this approach is 0.1. GO and Pathway analysis were conducted using WebGestalt [59].

### ^13^C tracer experiments

For tracer experiments, culture media was refreshed 4 h before cell harvest in order to avoid nutrient depletion. Glucose- and glutamine-free medium (DMEM, Genaxxon Bioscience, Ulm, Germany) was supplemented with the corresponding amount of lysine (146 mg/L) and arginine (84 mg/L) as well as ^13^C-labelled substrates: either U-^13^C_6_-glucose and ^12^C_5_-glutamine or ^12^C_6_-glucose and U-^13^C_5_-glutamine. For the U-^13^C_1_-sodium bicarbonate tracer experiment, glucose- and sodium bicarbonate-free media (XF Assay Medium Modified DMEM, Seahorse Bioscience, Copenhagen, Denmark) was supplemented with U-^13^C_1_-sodium bicarbonate (3.7 g/L), glucose (4.5 g/L) and glutamine (6 mM). U-^13^C_6_-glucose was purchased from Campro Scientific (Berlin, Germany), U-^13^C_5_-glutamine from Cambridge Isotope Laboratories (Andover, USA) and U-^13^C_1_-sodium bicarbonate from Isotec™ (Miamisburg, USA).

### Metabolite extraction and pulsed stable isotopic resolved metabolomics

All tracer experiments were performed in triplicate and a labelling period of 7 min for U-^13^C_6_-glucose and 15 min for U-^13^C_5_-glutamine was used (exceptions stated otherwise). After addition of tracer medium, cells were rapidly washed with a washing solution (5 mM HEPES, 3 mM KCl and 140 mM NaCl supplemented with the corresponding isotopes of ^13^C-glucose and ^13^C-glutamine). Cellular metabolism was quenched by addition of 5 mL ice-cold 50% methanol containing cinnamic acid as an internal standard (4 μg/ml). Cells were transferred to a reaction tube containing chloroform (total of 0.2 mL chloroform per 1.0 mL 50% methanol), vortexed, snap-frozen in liquid nitrogen and either stored at −80°C or thawed and shaken for at least 1 h at 750 rpm at 4°C. Phase separation was achieved by centrifuging at 7197 g for 10 min at room temperature. Polar and apolar phases were dried overnight in a vacuum concentrator (RVC 2–33 CD Plus coupled to a freeze dryer ALPHA 2–4 LD Plus, Christ, Germany). Tracer experiments with U-^13^C_1_-sodium bicarbonate were performed applying 20 min labelling time in AGS and HEK293 cells.

### Derivatization and GC/MS measurements

After metabolite extraction in 20% methanol and shaking at RT for 45 min, samples were dried overnight and prepared for GC-MS analysis as described [26]. Samples were analyzed in technical duplicates. The GC-MS chromatograms were processed with the ChromaTOF software (Version 4.42, LECO). A modified Golm metabolome database (GMD; http://gmd.mpimp-golm.mpg.de) version was used to identify substances with respect to spectra similarity and identical retention index. In addition, peaks were manually checked in comparison to an in-house identification mix containing the metabolites of interest. Data matrices for relative quantification and isotope patterns were extracted from the mass spectra exported files using the MetMax software [60]. The derivatization agent MBTSTFA + 1% TBDMCS (Sigma-Aldrich) was used as described [32] except for the sonication at the beginning. Samples were shaken at 750 rpm and the same n-alkane-standard was added to MBTSTFA + 1% TBDMCS. As identification standard, 10 μg of pure citrate were processed in the same way to obtain retention time and peak information.

### Data analysis of metabolomics

Each MetMax-extracted metabolite area information was normalized to the internal standard cinnamic acid before further statistical analysis was performed either in R [61] and RStudio (Version 0.96) with different packages as ggplot2 (Version 0.9.3), reshape2 (Version 1.2.2), stringr (Version 0.6.2) and plyr (Version 1.8) or in Microsoft Excel. For calculation of the label incorporation, MetMax was used to extract the corresponding metabolite intensities for defined isotopic mass ranges in combination with the retention index. Intensities of one identification standard were used as a reference for „unlabeled“ metabolites (reflecting the natural ^13^C abundance) in order to estimate the label incorporation into the metabolite of interest as described previously [62].

### Gene expression analysis of gastric cancer cell lines

Expression data from 37 gastric cancer cell lines (GSE22183) were normalized (‘RMA’ function; ‘affy’ package R v.3.1.0) and log2 transformed. Summarization was done by taking the median log2 expression of same-gene probes. To obtain “up-regulated” and “down-regulated” samples for the respective genes, the median gene profile was subtracted from the summarized gene expression for each sample. To examine potential co-expression patterns and/or patterns of expression mutual exclusivity, log2 gene expression for *HIF1A*, *MYC* and *ANXA1* across the cell lines were z-score-transformed and clustered using a euclidean-distance-based hierarchical clustering approach. Pearson's correlation analyses were also performed. Finally, hypothesis testing was carried out (cor.test function; ‘stats’ package R v.3.1.0) so as to estimate the association between paired genes and compute a test of the correlation value being zero.

### Immunohistochemistry

Immunohistochemical detection and analysis of HIF-1α and was performed according to our previously published protocol [15]. Annexin A1 was detected with a mouse monoclonal antibody (BD Biosciences) at a dilution of 1:100 after antigen retrieval consisting of 5 minutes heating in a pressure cooker. Tissue microarrays were constructed from 397 human gastric cancer samples using the Beecher manual TMA machine sampling from an area with the highest tumor cell density using three 0.6 mm tumor cores per case. All patients underwent surgical resection at the Leeds General Infirmary, UK, between 1968 and 2004. None of the patients received pre-operative chemotherapy or chemoradiotherapy.

### Statistical analysis

Shown are means ± SEM of at least three independent experiments. Statistical analysis was performed by unpaired Student's *t* test. Differences were considered statistically significant at *P* < 0.05.

### Data deposition

The microarray data from this publication have been submitted to the NCBI Gene Expression Omnibus (GEO) database (www.ncbi.nlm.nih.gov/geo) and assigned the identifier GSE57200.

## SUPPLEMENTARY MATERIALS AND FIGURES


