# Supplementary material for: Annexin A1 sustains tumor metabolism and cellular proliferation upon stable loss of HIF1A
Source: Oncotarget. 2015 Dec 29;7(6):6693–710. doi: 10.18632/oncotarget.6793 (PMC4872743; doi:10.18632/oncotarget.6793)
Supplement: Supplementary file 1 [file oncotarget-07-6693-s001.pdf]

Annexin A1 sustains tumor metabolism and cellular proliferation upon stable loss of HIF1A

Supplementary Materials

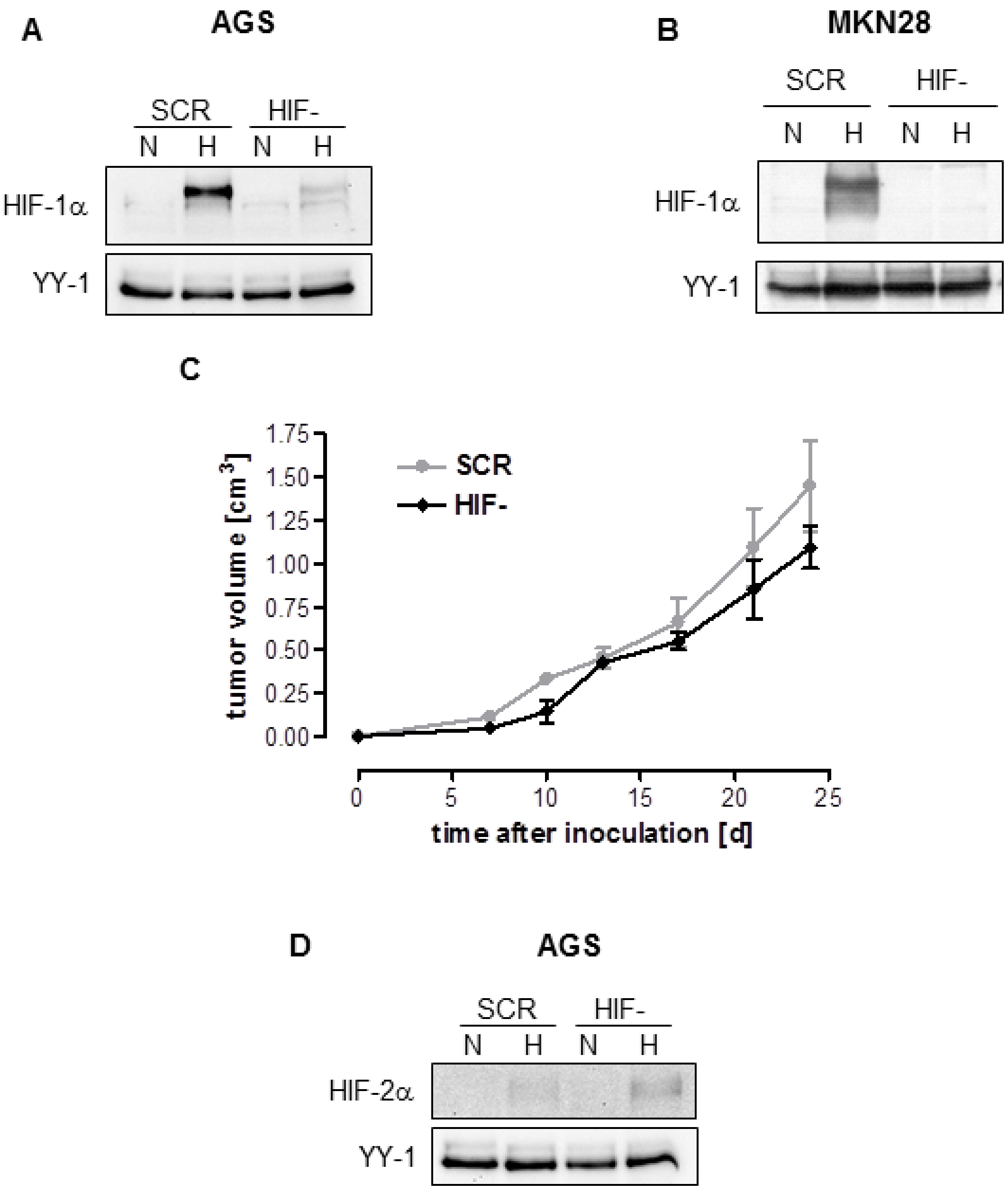

Supplementary Figure S1: Functional characterization of stable HIF-1a inhibition in gastric cancer cell lines AGS and MKN28. (**A, B**) AGS and MKN28 cells stably expressing shRNA against HIF 1α (HIF-) or scrambled control shRNA (SCR) were cultivated for 16 h under normoxia (N) or 1% oxygen (hypoxia (H)), and HIF-1α protein levels were analyzed by immunoblot, with YY-1 serving as nuclear loading control. (**C**) Xenograft growth of the human gastric cancer cell line MKN28. Subcutaneous tumors were generated by injecting 107 HIF-1α-deficient (HIF-) or control (SCR) MKN28 cells into the flanks of nude mice. Results are mean ± SEM ($n = 3$). (**D**) AGS cells stably expressing shRNA against HIF 1α (HIF-) or scrambled control shRNA (SCR) were cultivated for 6 h under normoxia (N) or 1% oxygen (hypoxia (H)), and HIF-2α protein levels were analyzed by immunoblot, with YY-1 serving as nuclear loading control.

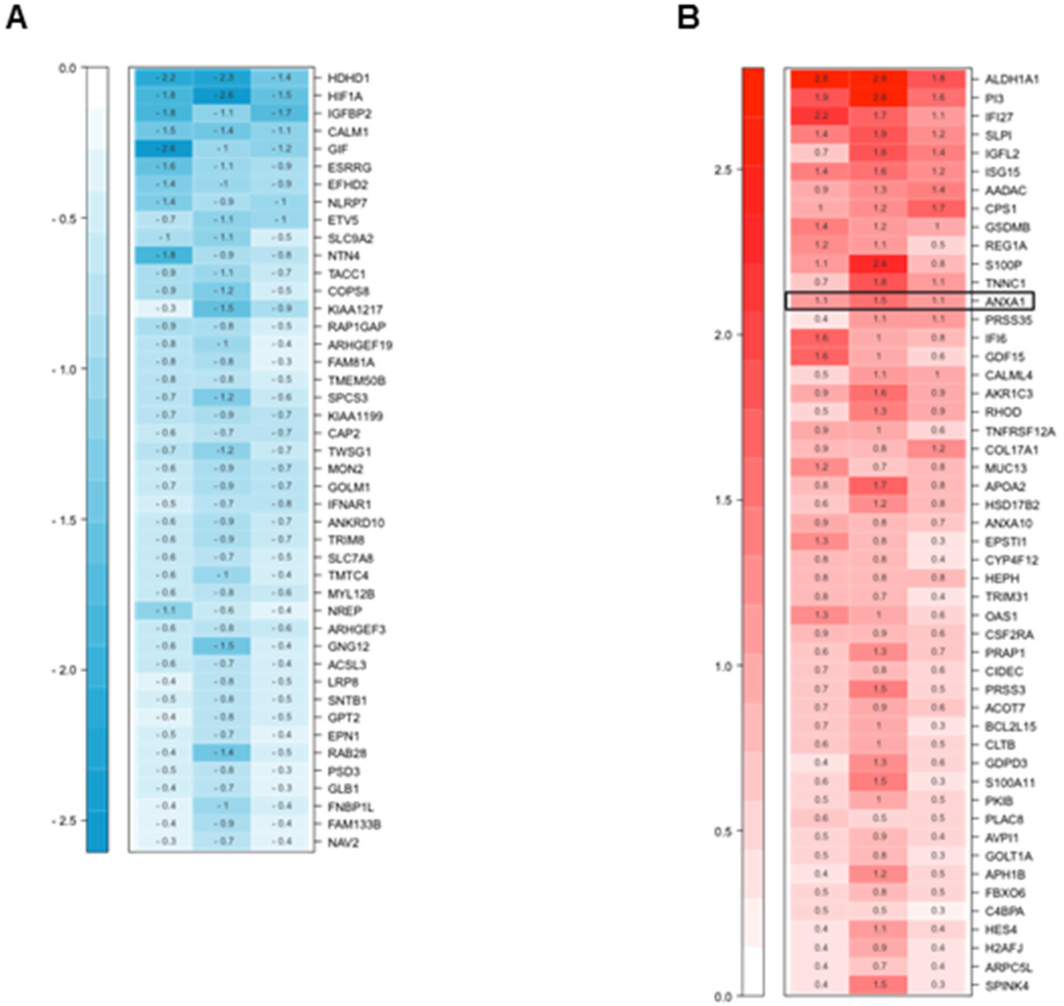

Supplementary Figure S2: Transcriptome analysis of HIF-1α-deficient AGS cells. (**A, B**) Heat map depicting the expression of down-regulated (**C**) and up-regulated (**D**) transcripts in HIF- cells compared to SCR cells. Columns represent biological replicates. Numbers within boxes indicate the fold difference in transcripts. Scale on the left show log2-based signals.

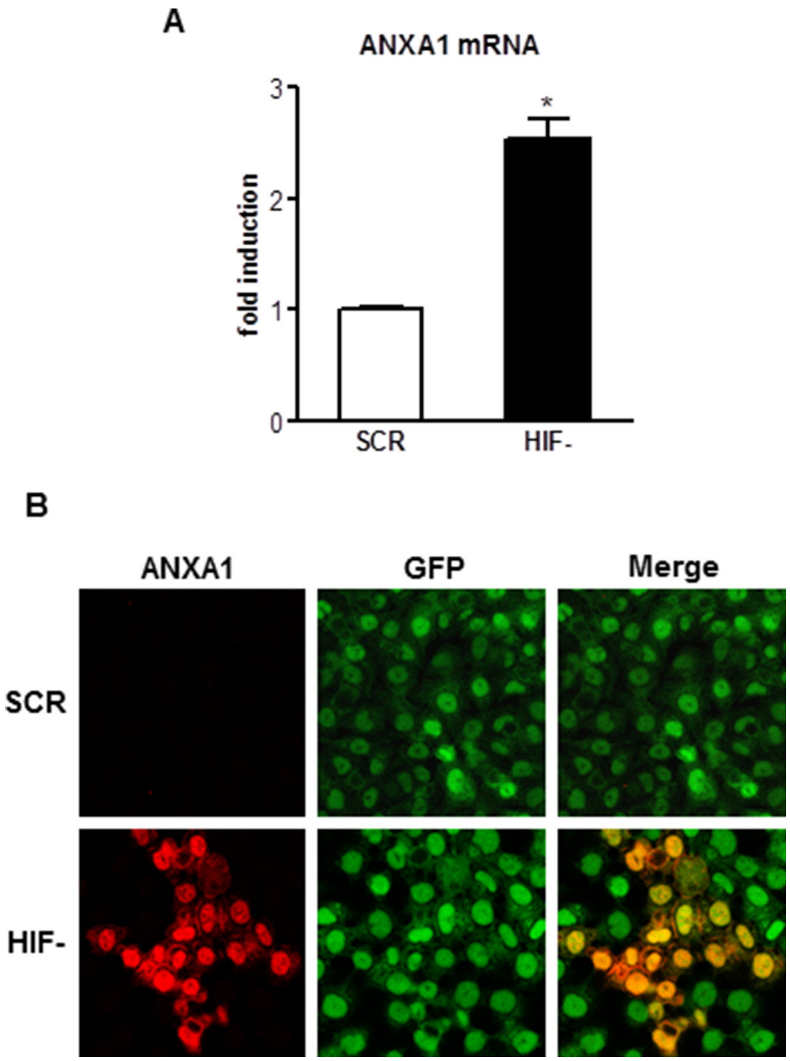

Supplementary Figure S3: Effect of HIF-1α inhibition on ANXA1 expression. (**A**) Expression of ANXA1 mRNA was measured relative to β-actin by quantitative real-time PCR. Transcription levels were expressed relative to that of control cells (SCR), set at 1.0. Values represent the mean ± SEM (**P* < 0.05). (**B**) Confocal immunofluorescent detection of ANXA1 in AGS SCR (top) and HIF-(bottom) AGS cells. Magnification x630.

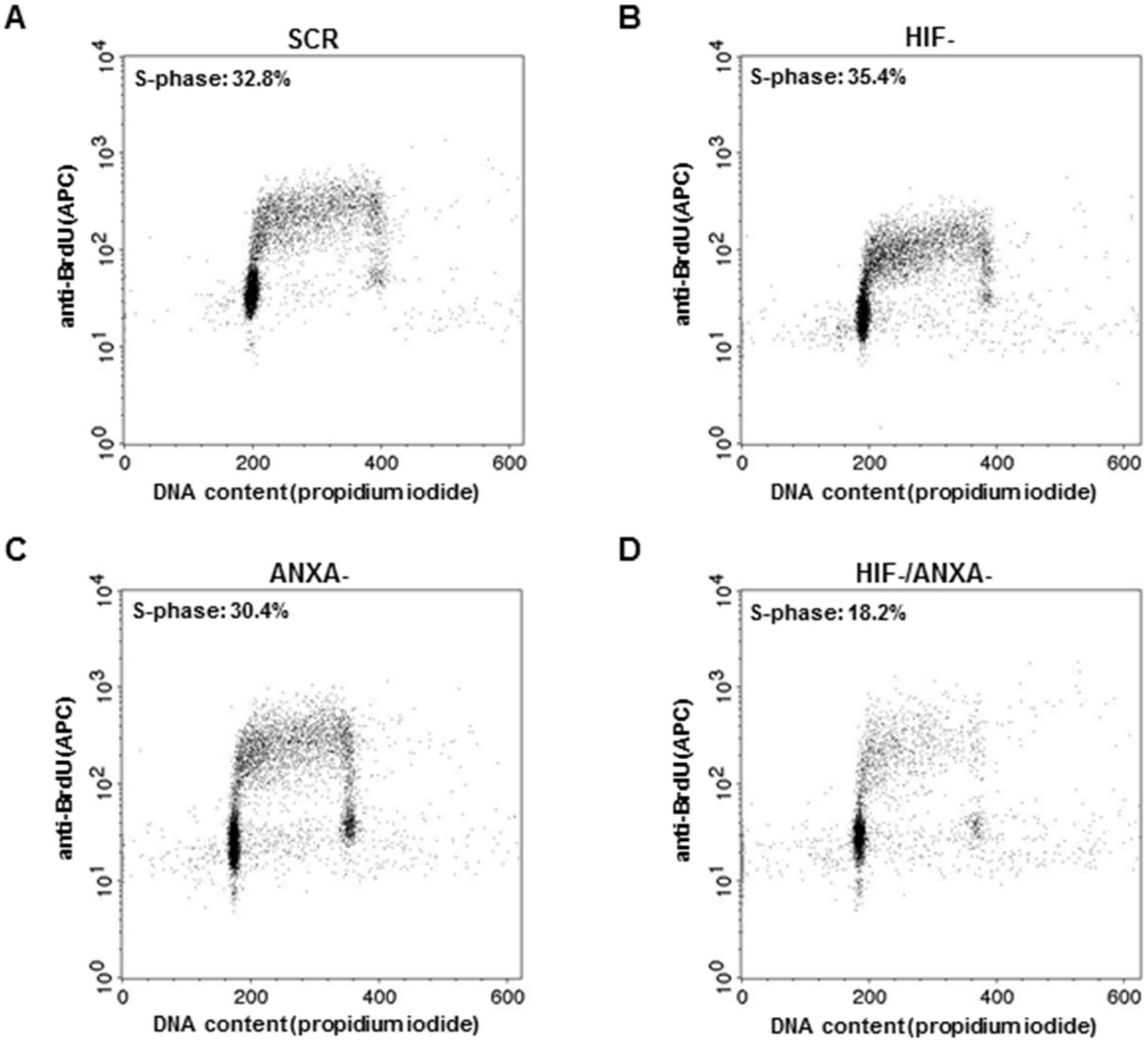

Supplementary Figure S4: Effects of combined inhibition of HIF-1α and ANXA1 on cell proliferation. (**A–D**) Rate of DNA synthesis was determined by BrdU and propidium iodide staining with subsequent FACS analysis. Shown are representative dot plots of control (SCR, A), HIF-1α-deficient (HIF-, B), ANXA1-deficient (ANXA-, C) and double knock-down (HIF-/ANXA-, D) AGS cells. The fraction of cells in S-phase is given as percentage of total cell number.

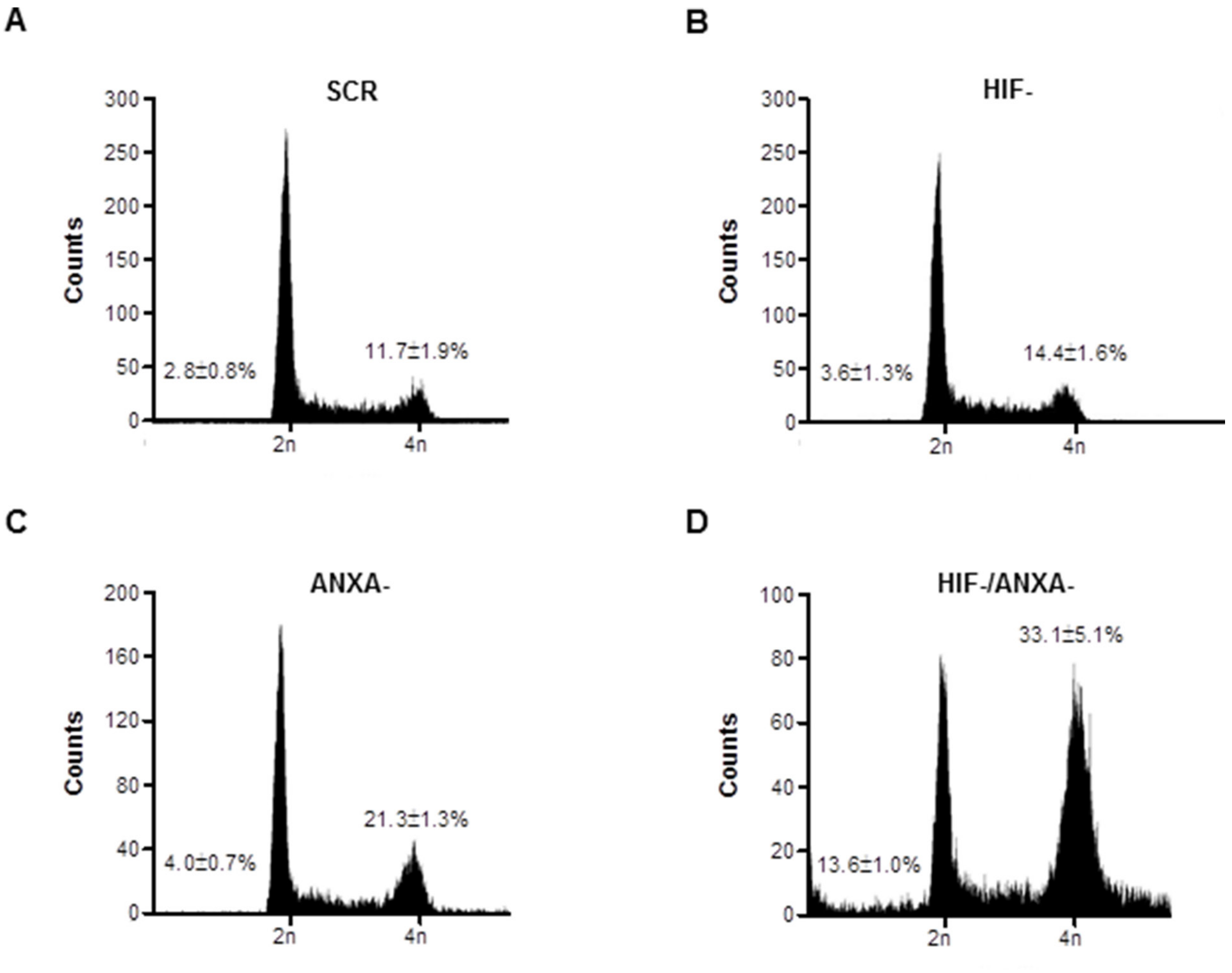

Supplementary Figure S5: Effects of combined inhibition of HIF-1a and ANXA1 on cell cycle distribution. (A–D) Cell cycle was determined by propidium iodide staining with subsequent FACS analysis. Shown are representative histograms of control (SCR, A), HIF-1α-deficient (HIF-, B), ANXA1-deficient (ANXA-, C) and double knock-down (HIF-/ANXA-, D) AGS cells. The fractions of cells in pre-G1 and G2/M phase are given as percentage of total cell number.

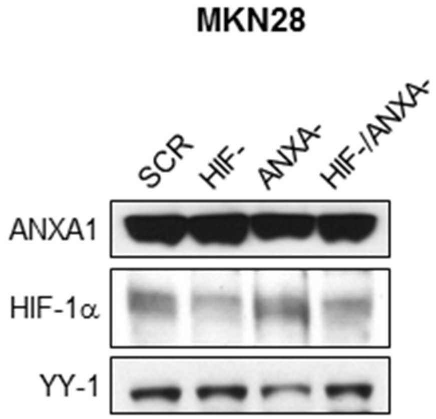

Supplementary Figure S6: Expression of ANXA1 and HIF-1α in MKN28 cells. Representative immunoblot analysis of ANXA1 and HIF-1α in control (SCR), HIF-1α-deficient (HIF-), ANXA1-deficient (ANXA-) and double knock-down (HIF-/ANXA-) MKN28 cells. Cells were passaged regularly and grown as monolayer cultures under recommended culture conditions for four weeks. YY-1 served as loading control.

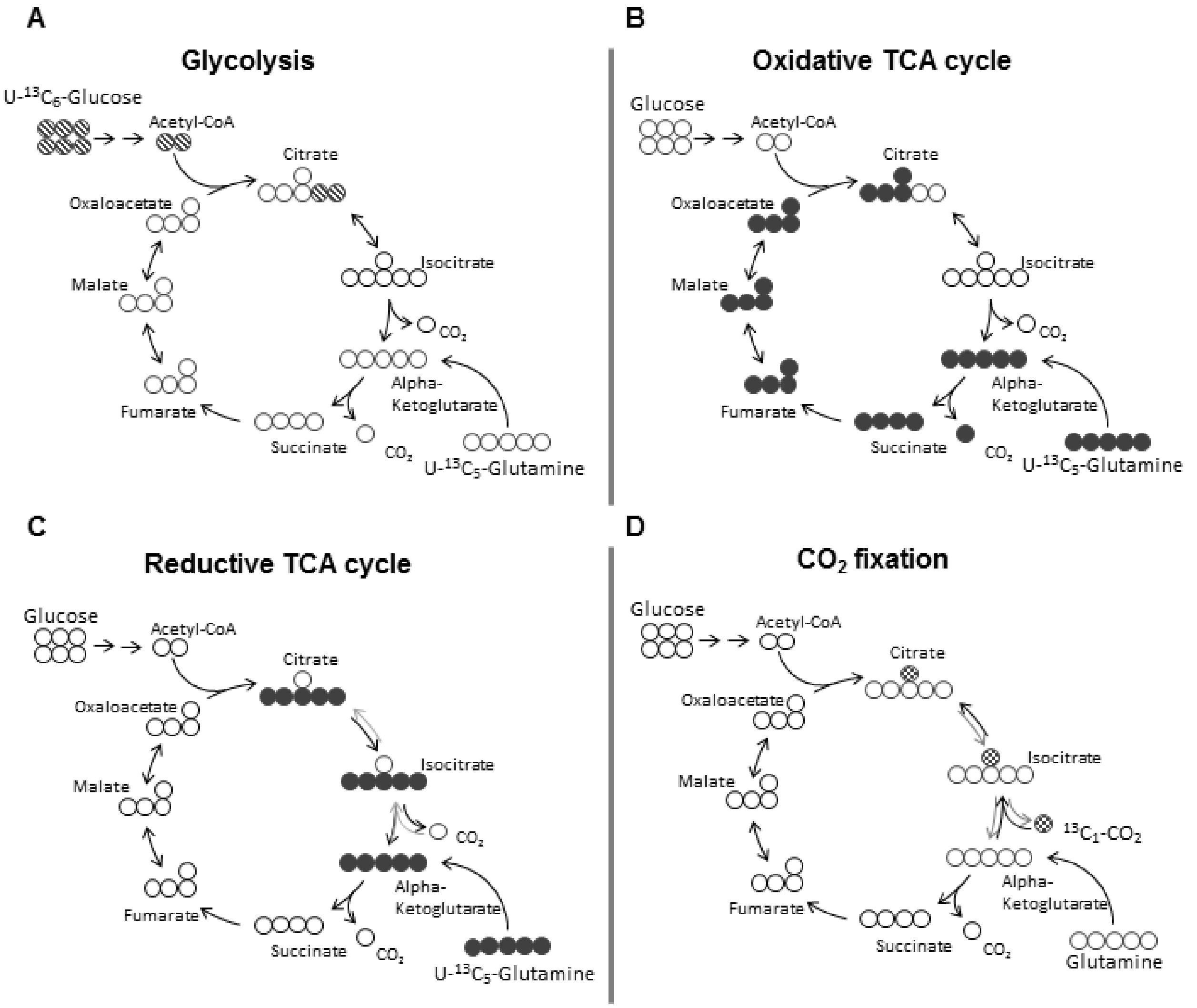

Supplementary Figure S7: Schematic representation of the carbon atom transitions into citrate with different 13C-labelled substrates. (**A–D**) Schematic representation of the carbon atom transitions into citrate during glycolysis (A), oxidative TCA metabolism (B), reductive TCA metabolism (C) and CO2 fixation (D) after three different labelling strategies: U-^{13}C6-glucose, U-^{13}C5-glutamine and ^{13}C1-carbon dioxide. These labelling strategies result in different mass isotopomers of citrate originating from these distinct uniformly ^{13}C-substrates.

A

citrate mass isotopomer	nutrient source	metabolism	observable GC-MS fragments	label incorporation in
	U-$^{13}C_6$-glucose	oxidative	C5: C6:	m+2
	U-$^{13}C_5$-glutamine	oxidative	C5: C6:	m+3 | m+4
	U-$^{13}C_5$-glutamine	reductive	C5: C6:	m+5
	U-$^{13}C_1$-sodium bicarbonate	reductive	C5: C6:	------ | m+1

B

[m/z]	MSTFA		MBTSFTA
Mass shift	C5-Fragment	C6-Fragment	C6-Fragment
m0	273	375	459
m+1	274	376	460
m+2	275	377	461
m+3	276	378	462
m+4	277	379	463
m+5	278	380	464
m+6	-	381	465

C

		MSTFA			
		[m/z]	Labeling source		
Metabolite	mass fragment	^{12}C	^{13}C-glucose	^{13}C-glutamine	^{13}C-sodium bicarbonate
Alanine	C2	188	190	-	-
Citrate	C5	273	275	276, 278	274
Citrate	C6	375	377	380	376
Fructose-6-Phosphate	C3	217	220	-	-
Fumarate	C3	245	247	249	-
Glucose-6-phosphate	C3	217	220	-	-
Glutamate	C4	246	-	250	-
Glycerol-3-phosphate	C3	357	359	-	-
Alpha-Ketoglutarate	C3	198	200	203	-
Lactate	C2	219	222	-	-
Malate	C3	233	235	236	-
3-Phosphoglycerate	C3	357	359	-	-
Pyruvate	C3	174	177	-	-
Succinate	C4	247	249	251	-

		MBTSTFA			
			Labeling source		
Metabolite	mass fragment	^{12}C	^{13}C-glucose	^{13}C-glutamine	^{13}C-sodium bicarbonate
Citrate	C6	459	-	-	460

Supplementary Figure S8: Different mass isotopomers at their corresponding m/z values detectable by GC-MS depending on the tracer substrate. (**A**) Table showing four different mass isotopomers (MI) of citrate and the corresponding source of labelled substrates and metabolism route, illustrating the observable metabolite mass fragments (C5 or C6: either five or six carbon backbone) via GC-MS and the corresponding detectable mass shifts of the MIs. (**B**) Table showing the different mass shifts of citrate and the corresponding m/z of the different GC-MS fragments. The m/z for citrate depends on the derivatization agent used. (**C**) Table depicting in more detail which MIs and fragments where used for calculation of the different labelled quantities of metabolites used in Figure 3. Additionally, it shows the m/z values corresponding to the different labelling strategies and derivatization agents used.

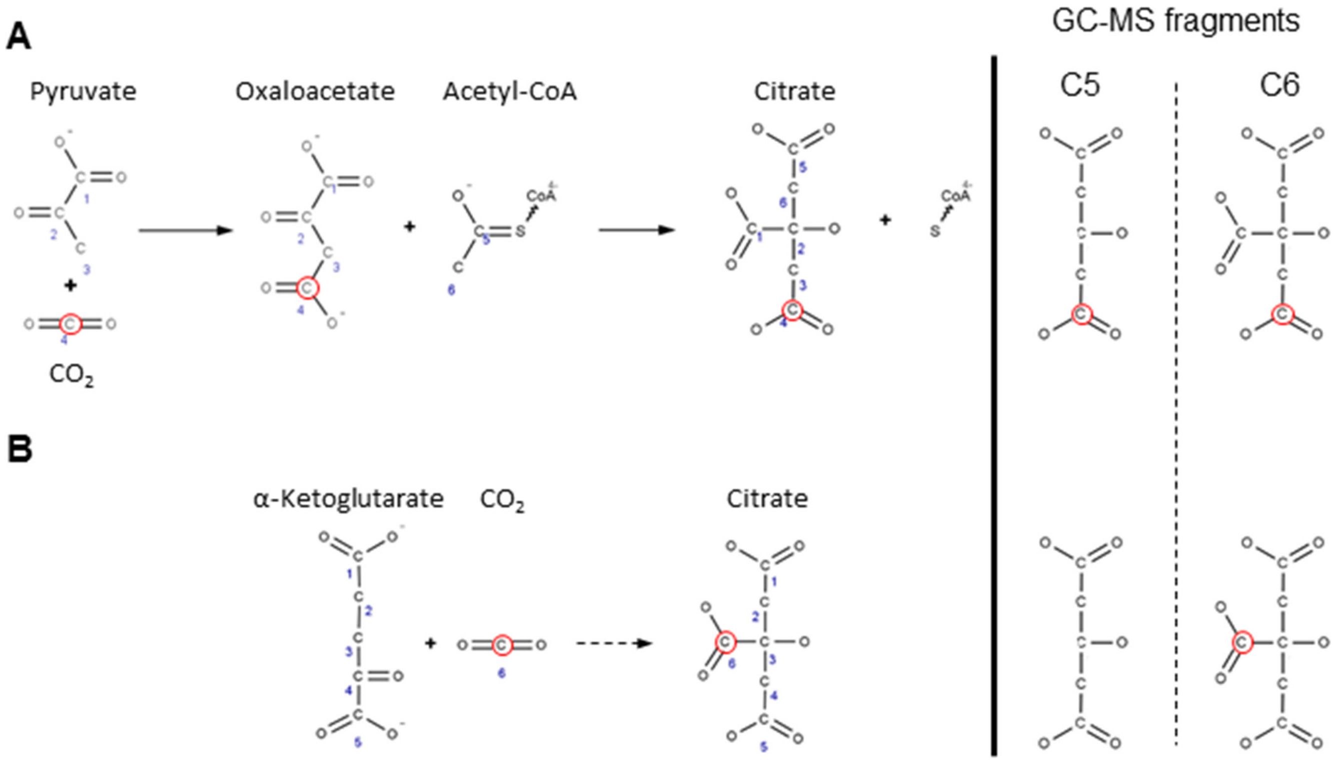

Supplementary Figure S9: $^{13}CO_2$ atom position via carboxylation in the central carbon metabolism. (**A**) Anaplerotic reaction from pyruvate to oxaloacetate by pyruvate carboxylase. The carbon atom introduced into citrate via carbon dioxide is marked with a red circle. Even though citrate is a symmetric molecule, both precursors (oxaloacetate and acetyl-CoA) are asymmetric and react in a position-specific manner to produce citrate. Thus, the positional information of carbon atoms is conserved. The carboxylation of pyruvate introduces one carbon at position 4 of oxaloacetate. That integrates at position 4 of the citrate molecule (according to our nomenclature in A). (**B**) Assuming that the exact same biochemical reaction from citrate to α-ketoglutarate is taking place in a reverse manner for reductive TCA metabolism, the carbon atom introduced by the reductive carboxylation of α-ketoglutarate introduces a carbon atom at position 6 of the citrate molecule (according to our nomenclature in B). On the right side the observable mass isotopomers via the used GC-MS approach are shown. Both reactions introduce a m + 1 in the C6-fragment (m/z = 375; MSTFA derivatization). The C5-fragment (m/z 273; MSTFA derivatization) does not contain the carbon introduced via reductive carboxylation (Figure 4C). Reaction principles are taken from "Lehninger, principles of biochemistry", 5th edition, 2008, Freeman and company, New York, USA.

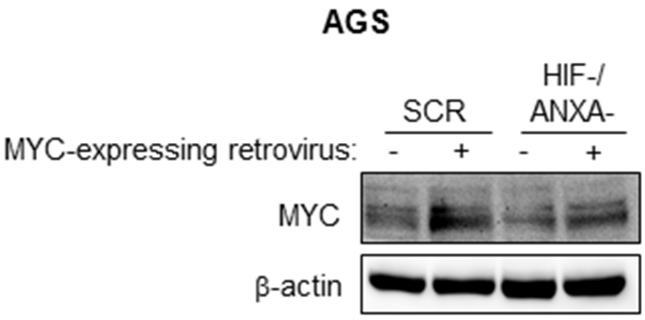

Supplementary Figure S10: Demonstration of the efficacy of the MYC-expression plasmid in AGS gastric cancer cells. AGS cells stably re-expressing MYC were generated by transduction with retroviruses containing human MYC as outlined in materials and methods. Whole cell lysates were isolated and MYC protein levels were analyzed by immunoblot, with β-actin as the loading control.

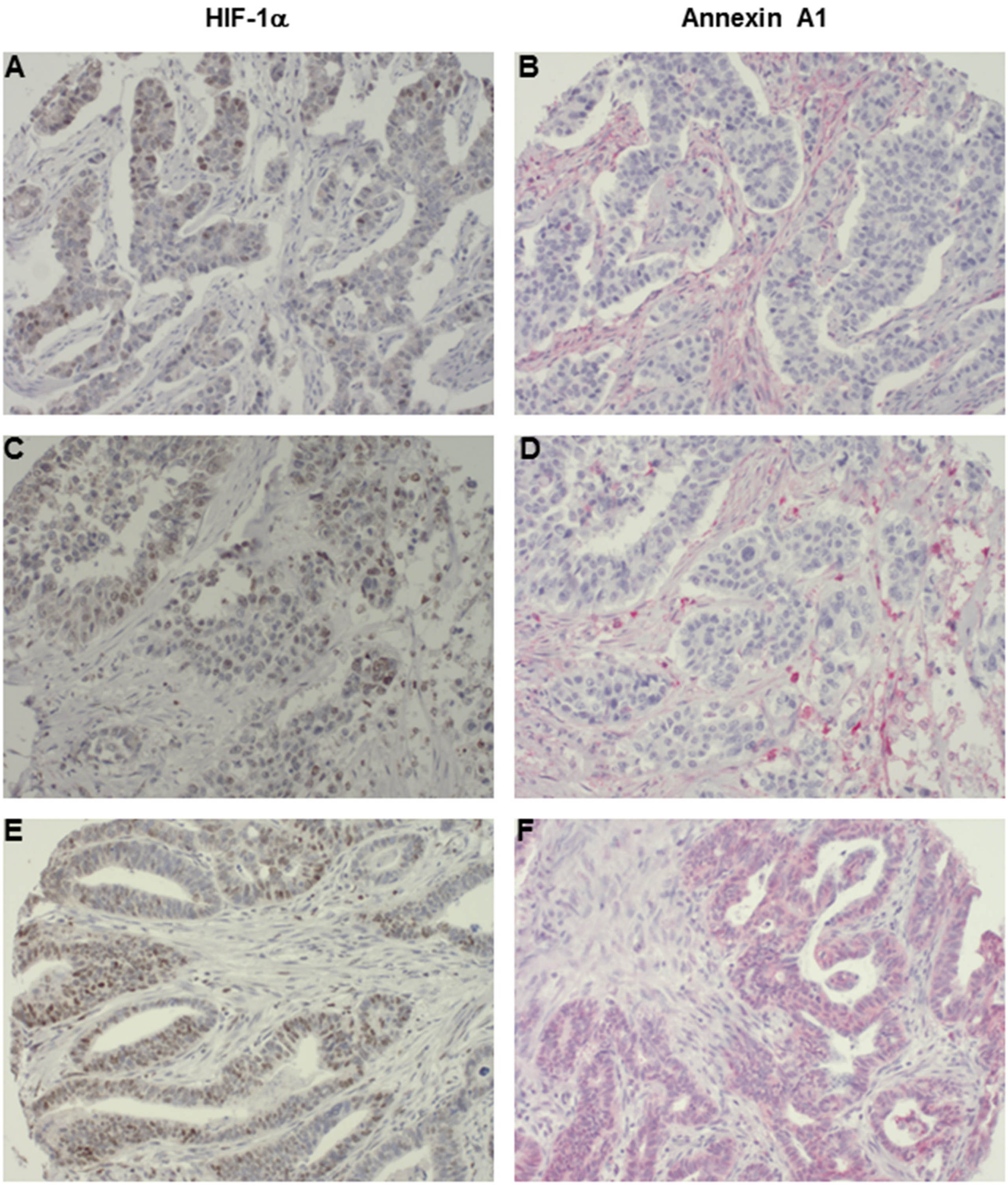

Supplementary Figure S11: Immunohistochemical analysis of HIF-1α and ANXA1 protein distribution in human gastric cancer. (**A–F**) HIF-1α (A,C,E; brown staining) and ANXA1 (B,D,F; red signal) protein was localized via immunohistochemistry with monospecific antibodies. Appreciating the function of HIF-1α as a transcription factor, only nuclear signal was considered specific. ANXA1 protein was detected in a specific manner both in the nucleus and in the cytoplasm of cancer cells. The majority of HIF-1α positive gastric cancer samples studied were ANXA1-negative (representative examples are shown in A, B, C and D). As outlined in the results section, 19.6% of HIF-1α-positive samples were also ANXA1-positive (representative example shown in E and F). Note the positivity of the tumor stroma for ANXA1 (B, D).

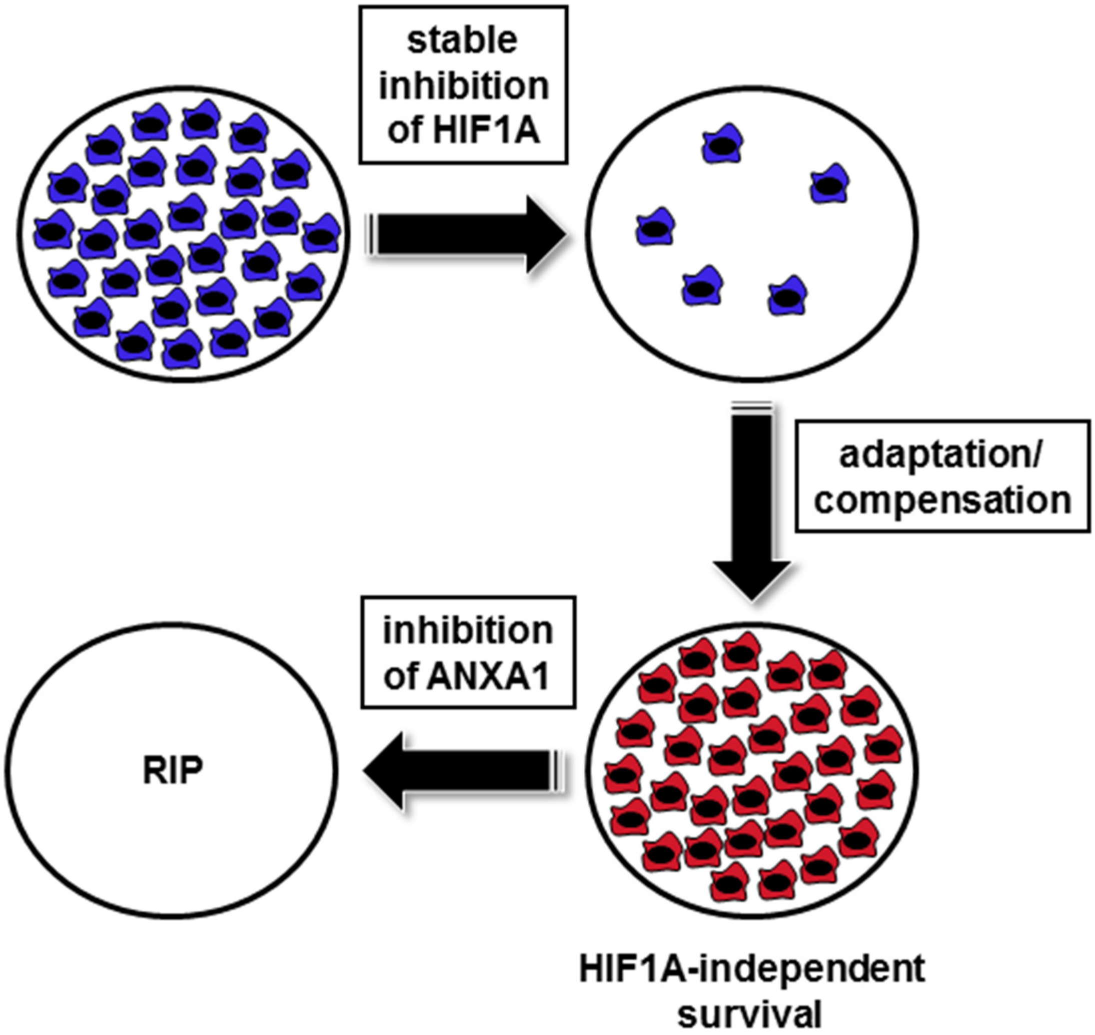

Supplementary Figure S12: Schematic representation of the induced essentiality between HIF1A and ANXA1.
